# Supplementary material for: Modelling the effects of bacterial cell state and spatial location on tuberculosis treatment: Insights from a hybrid multiscale cellular automaton model
Source: J Theor Biol. 2018 Jun 7;446:87–100. doi: 10.1016/j.jtbi.2018.03.006 (PMC5901892; doi:10.1016/j.jtbi.2018.03.006)
Supplement: Supplementary Data S1 — Supplementary Raw Research Data. This is open data under the CC BY license http://creativecommons.org/licenses/by/4.0/ [file mmc1.zip › Supplementary material/Tcellplots.pdf]

T cell plots

This file contains plots for the temporal dynamics of the T cells in simulations where the T cells were non-zero. This includes one simulation in the fixed scenario and 12 in the random scenario.

Fixed distribution

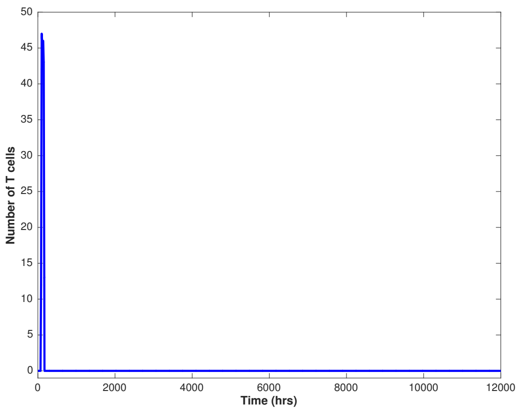

Simulation 20

Random distribution

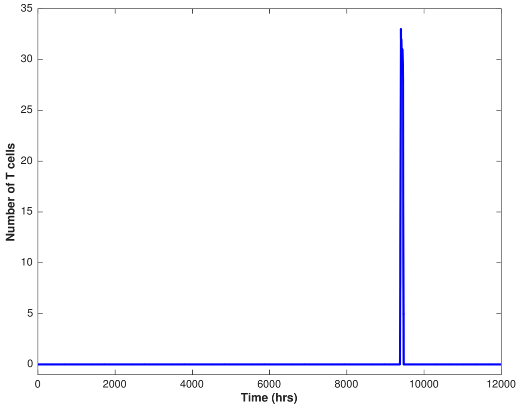

Simulation 6

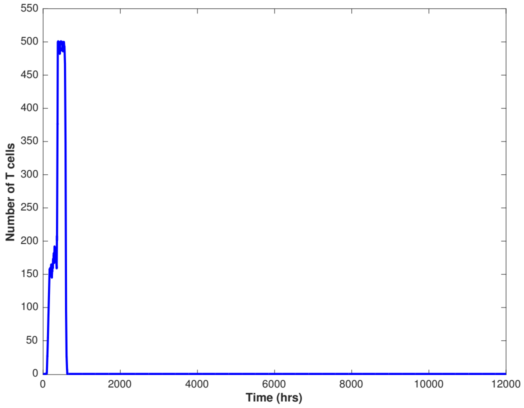

Simulation 9

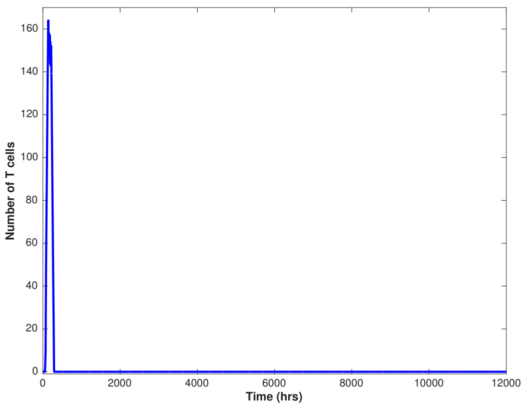

Simulation 15

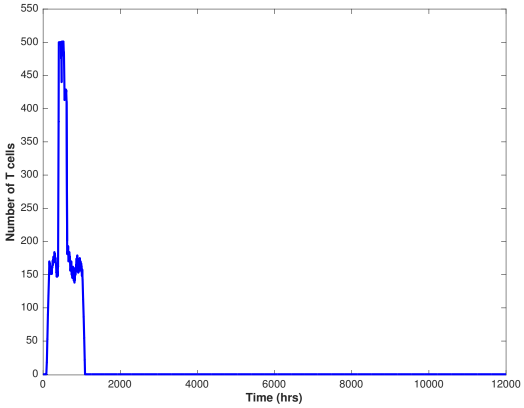

Simulation 22

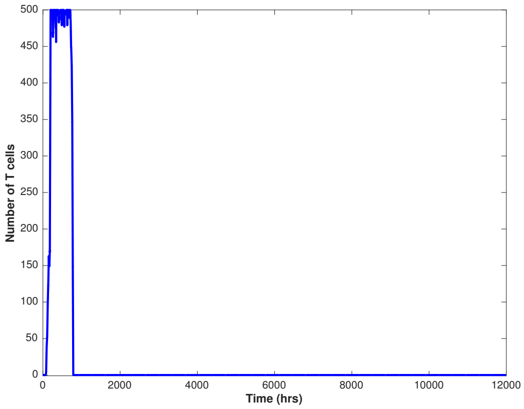

Simulation 31

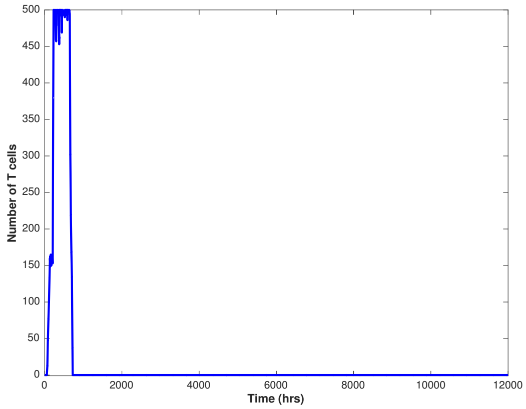

Simulation 48

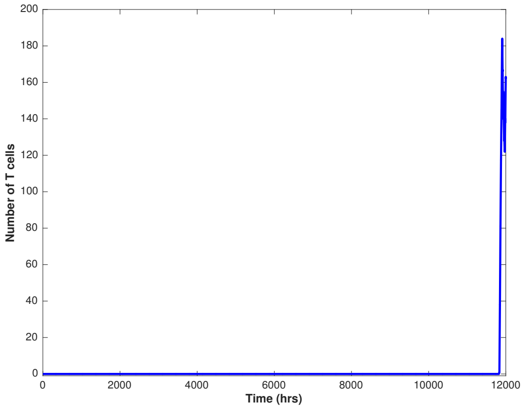

Simulation 60

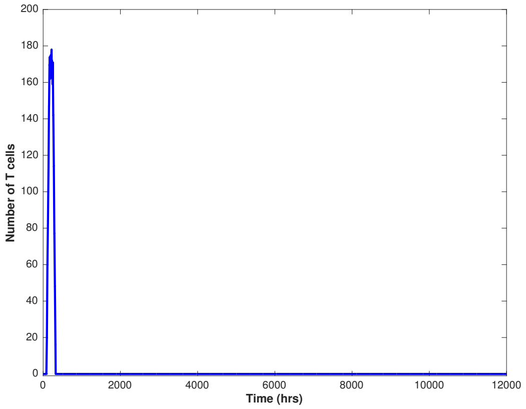

Simulation 71

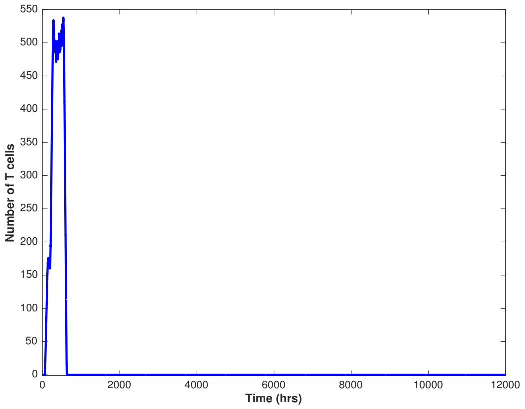

Simulation 82

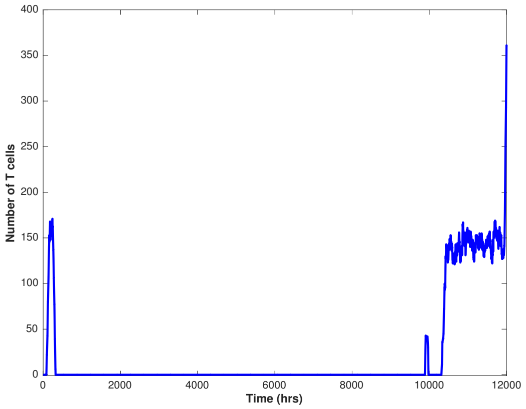

Simulation 88

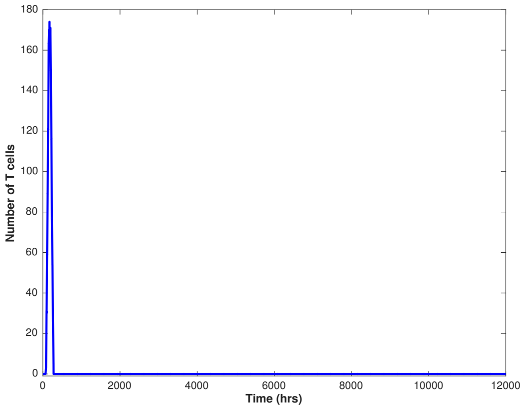

Simulation 89

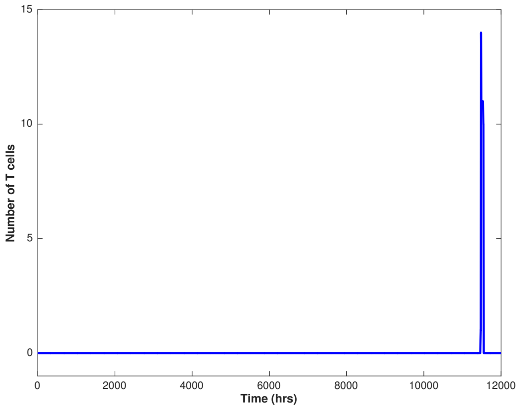

Simulation 90
