# Supplementary figures and images for: Modelling the effects of bacterial cell state and spatial location on tuberculosis treatment: Insights from a hybrid multiscale cellular automaton model
Source: J Theor Biol. 2018 Jun 7;446:87–100. doi: 10.1016/j.jtbi.2018.03.006 (PMC5901892; doi:10.1016/j.jtbi.2018.03.006)

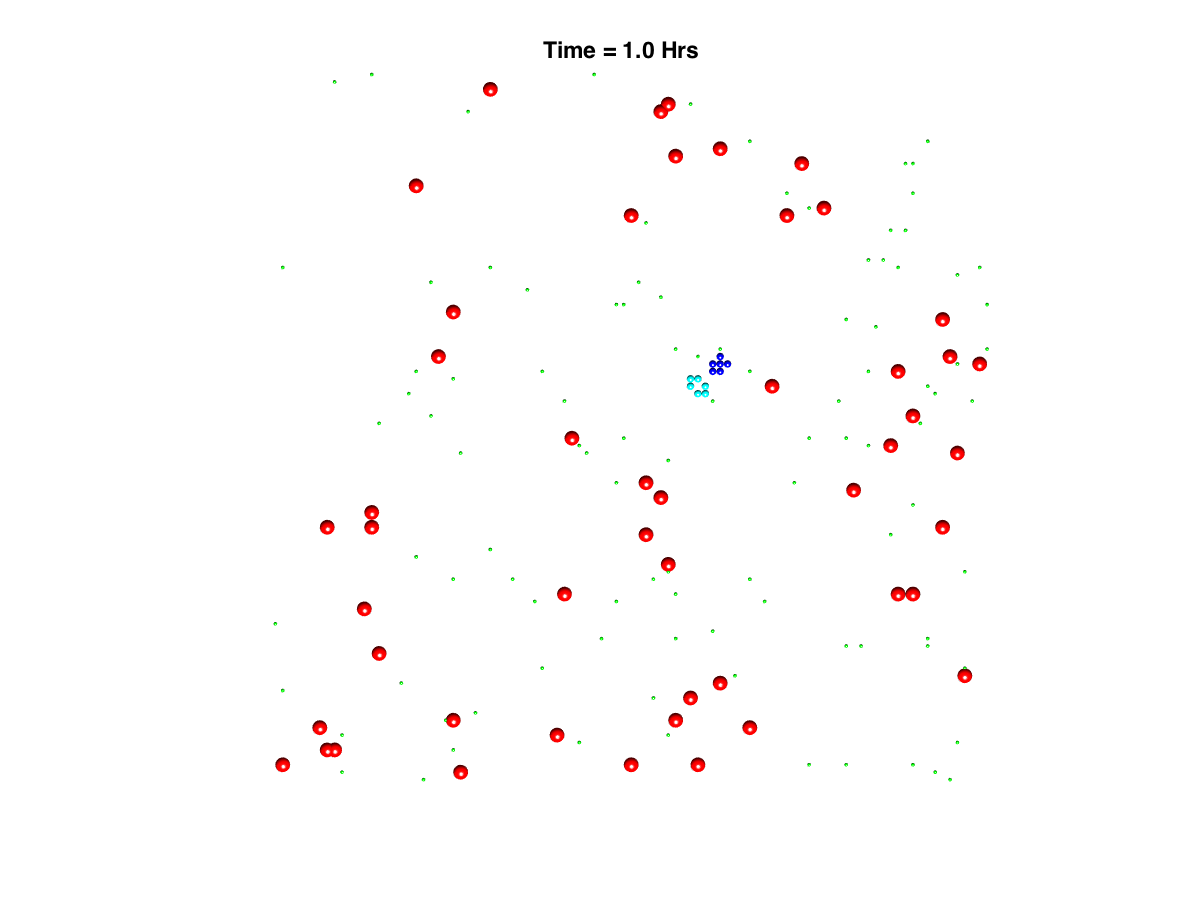

Supplement: Supplementary Data S1 — Supplementary Raw Research Data. This is open data under the CC BY license http://creativecommons.org/licenses/by/4.0/ [file mmc1.zip › Supplementary material/Figures 7-10/Figure10ai.png]

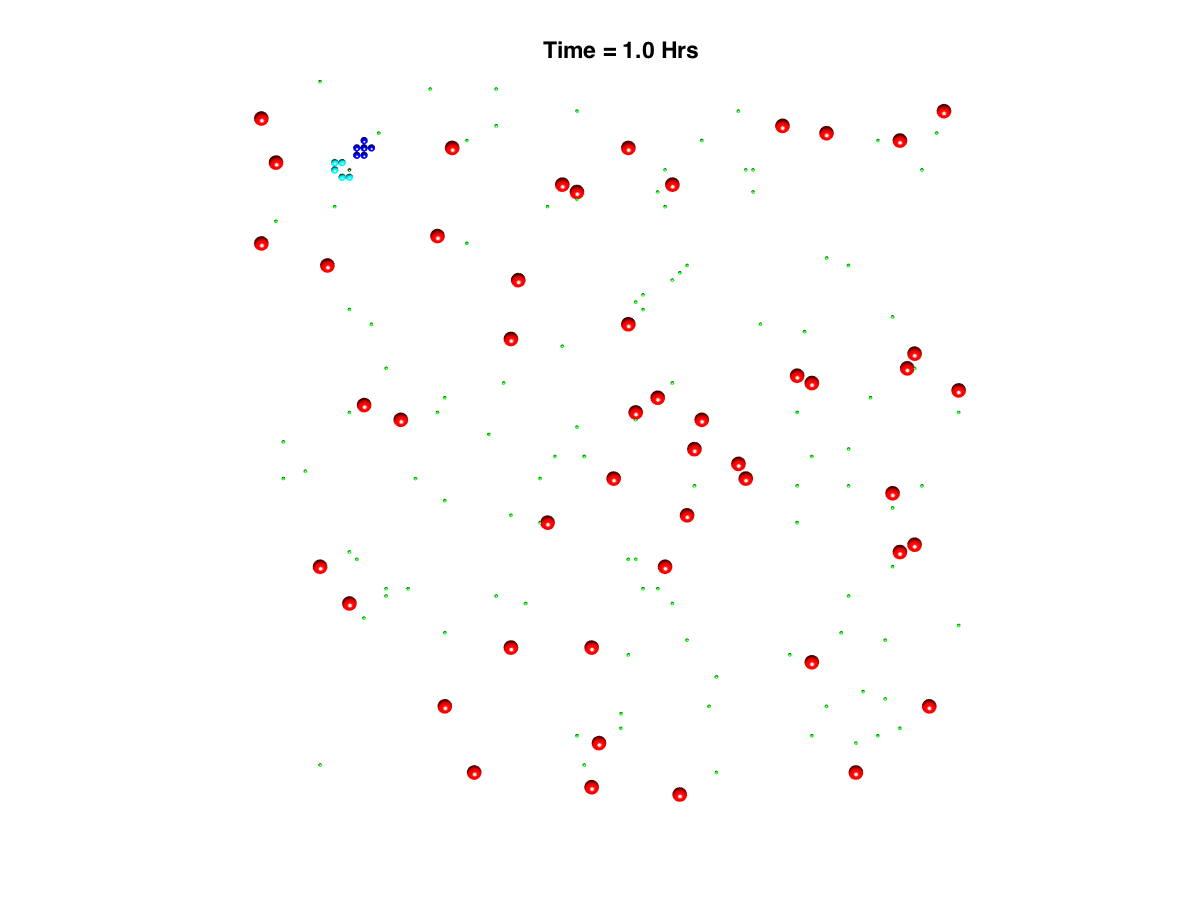

Supplement: Supplementary Data S1 — Supplementary Raw Research Data. This is open data under the CC BY license http://creativecommons.org/licenses/by/4.0/ [file mmc1.zip › Supplementary material/Figures 7-10/Figure10aii.png]

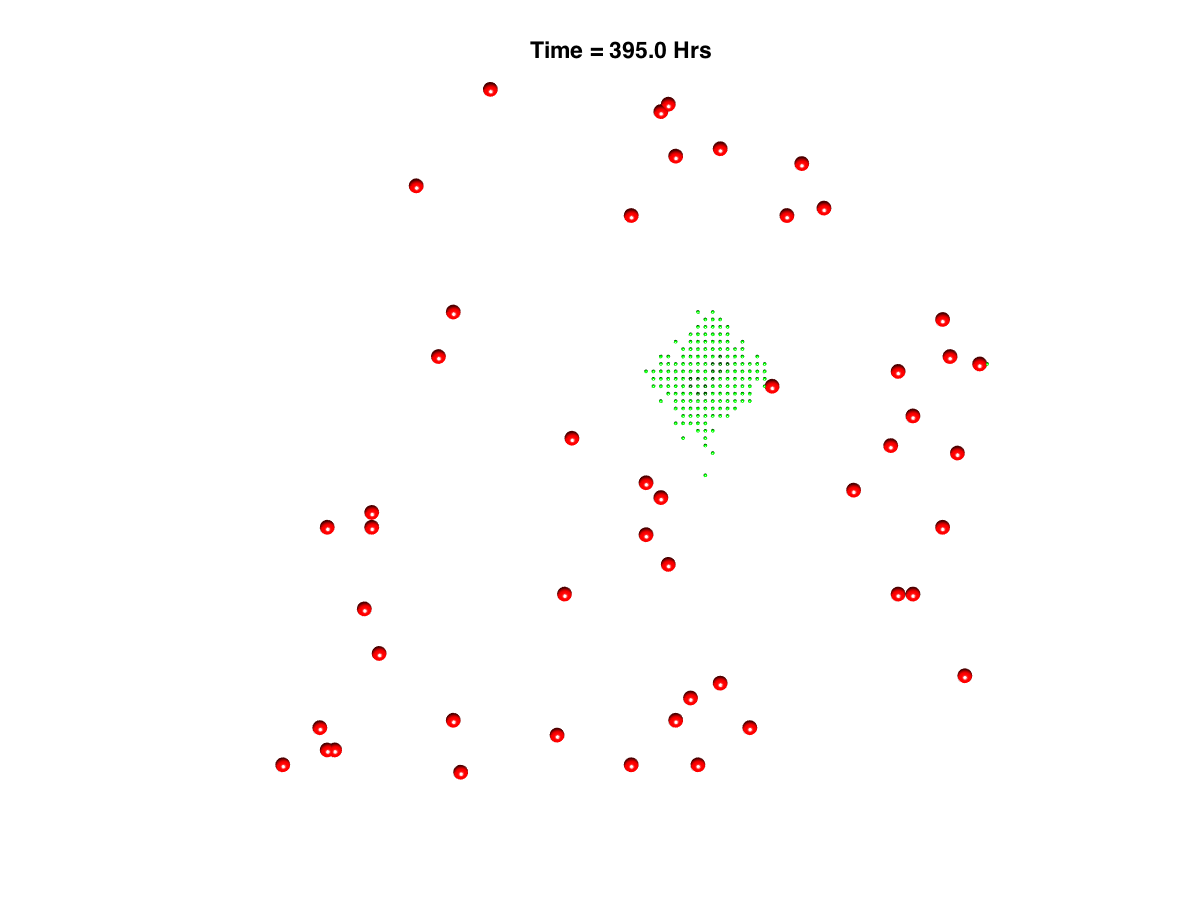

Supplement: Supplementary Data S1 — Supplementary Raw Research Data. This is open data under the CC BY license http://creativecommons.org/licenses/by/4.0/ [file mmc1.zip › Supplementary material/Figures 7-10/Figure10bi.png]

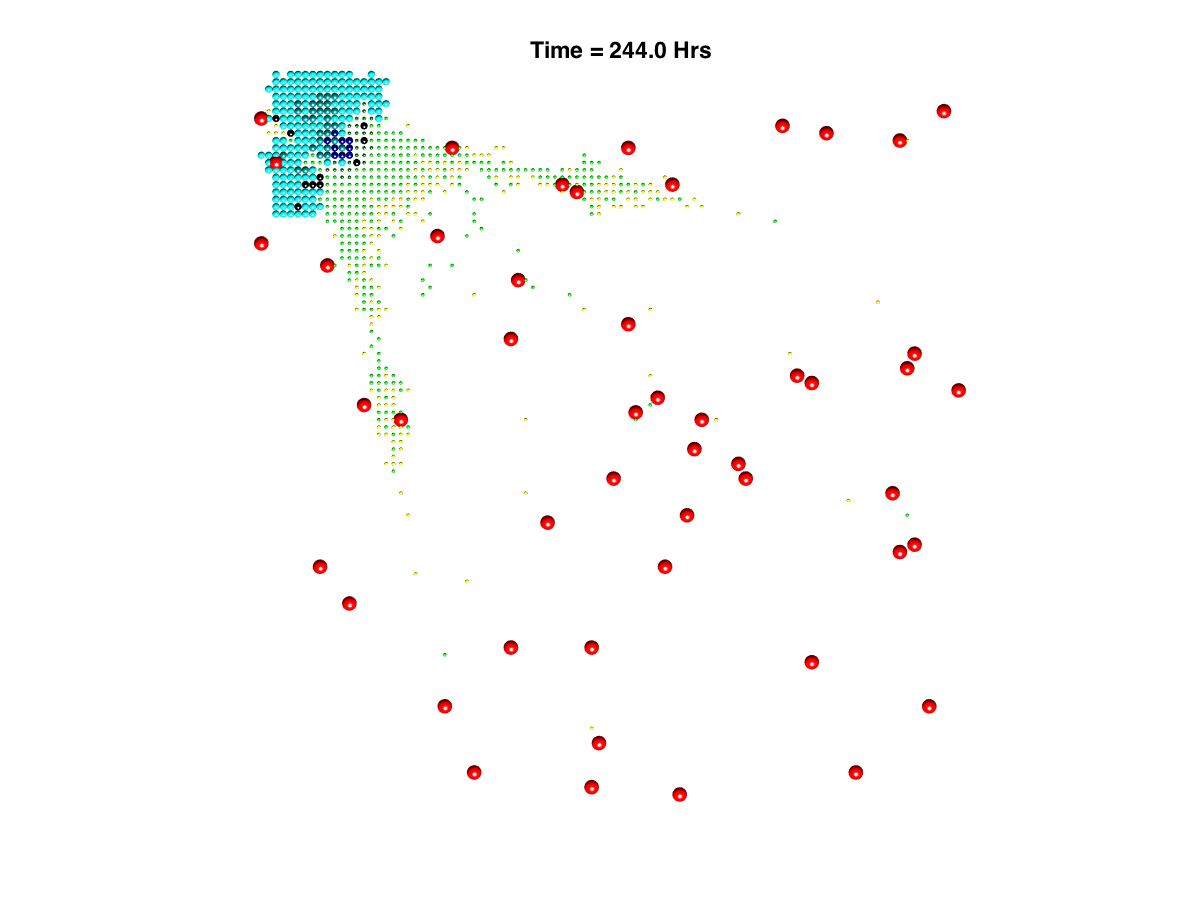

Supplement: Supplementary Data S1 — Supplementary Raw Research Data. This is open data under the CC BY license http://creativecommons.org/licenses/by/4.0/ [file mmc1.zip › Supplementary material/Figures 7-10/Figure10bii.png]

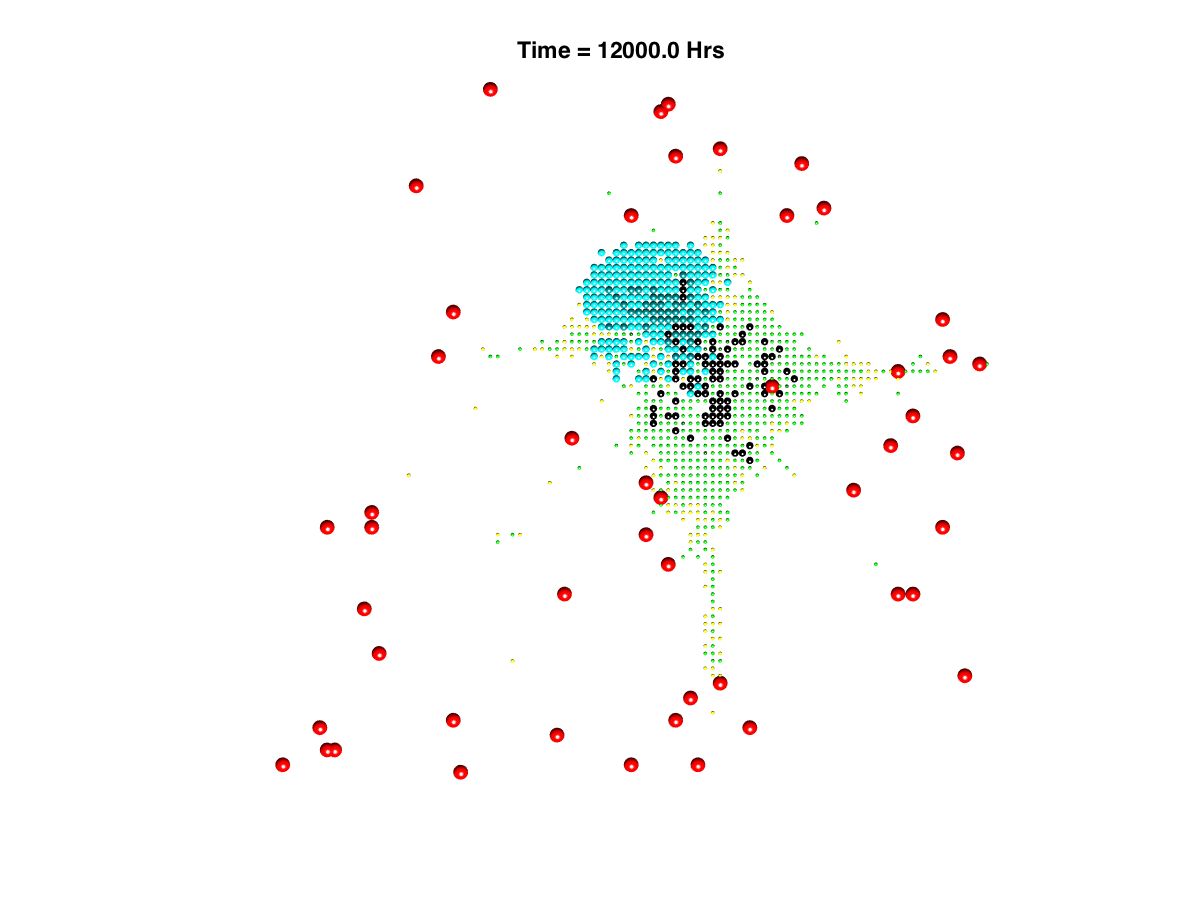

Supplement: Supplementary Data S1 — Supplementary Raw Research Data. This is open data under the CC BY license http://creativecommons.org/licenses/by/4.0/ [file mmc1.zip › Supplementary material/Figures 7-10/Figure10ci.png]

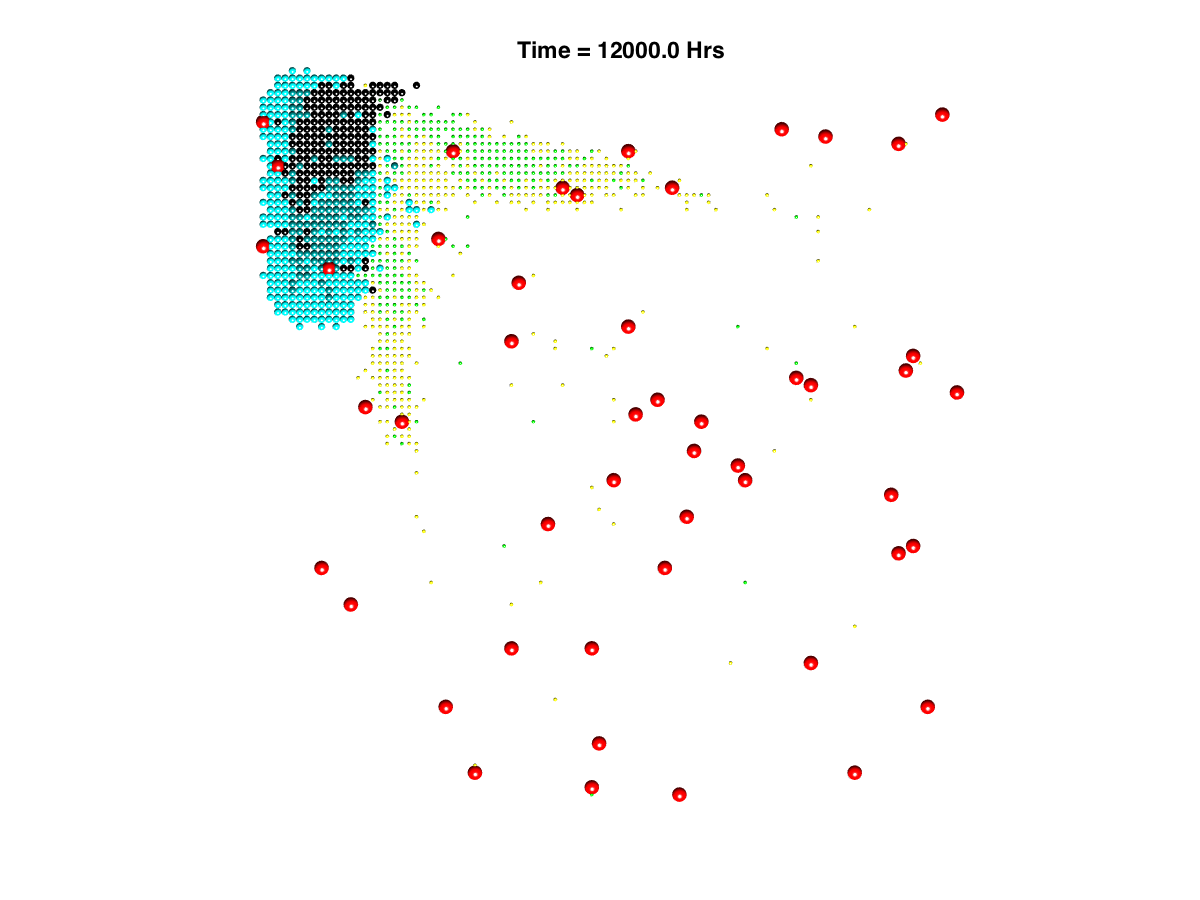

Supplement: Supplementary Data S1 — Supplementary Raw Research Data. This is open data under the CC BY license http://creativecommons.org/licenses/by/4.0/ [file mmc1.zip › Supplementary material/Figures 7-10/Figure10cii.png]

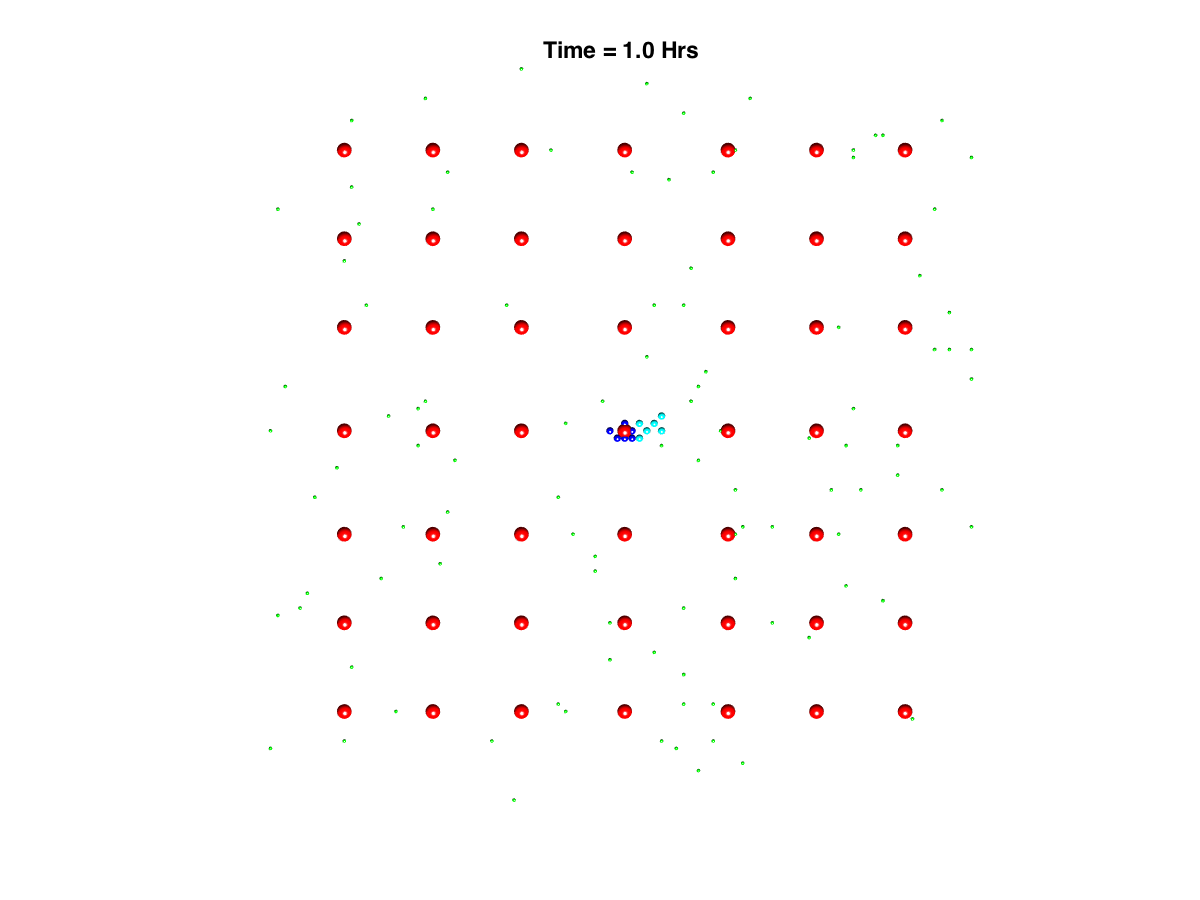

Supplement: Supplementary Data S1 — Supplementary Raw Research Data. This is open data under the CC BY license http://creativecommons.org/licenses/by/4.0/ [file mmc1.zip › Supplementary material/Figures 7-10/Figure7ai.png]

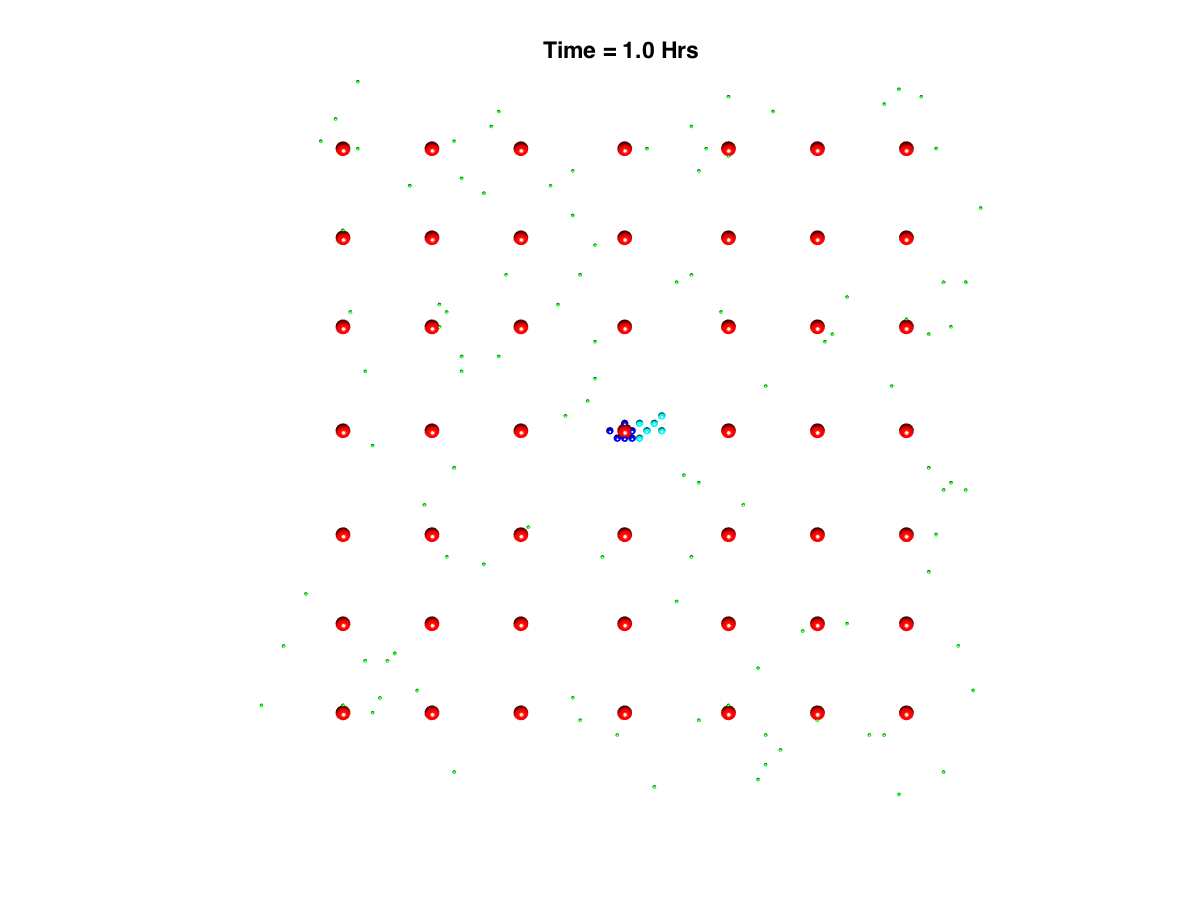

Supplement: Supplementary Data S1 — Supplementary Raw Research Data. This is open data under the CC BY license http://creativecommons.org/licenses/by/4.0/ [file mmc1.zip › Supplementary material/Figures 7-10/Figure7aii.png]

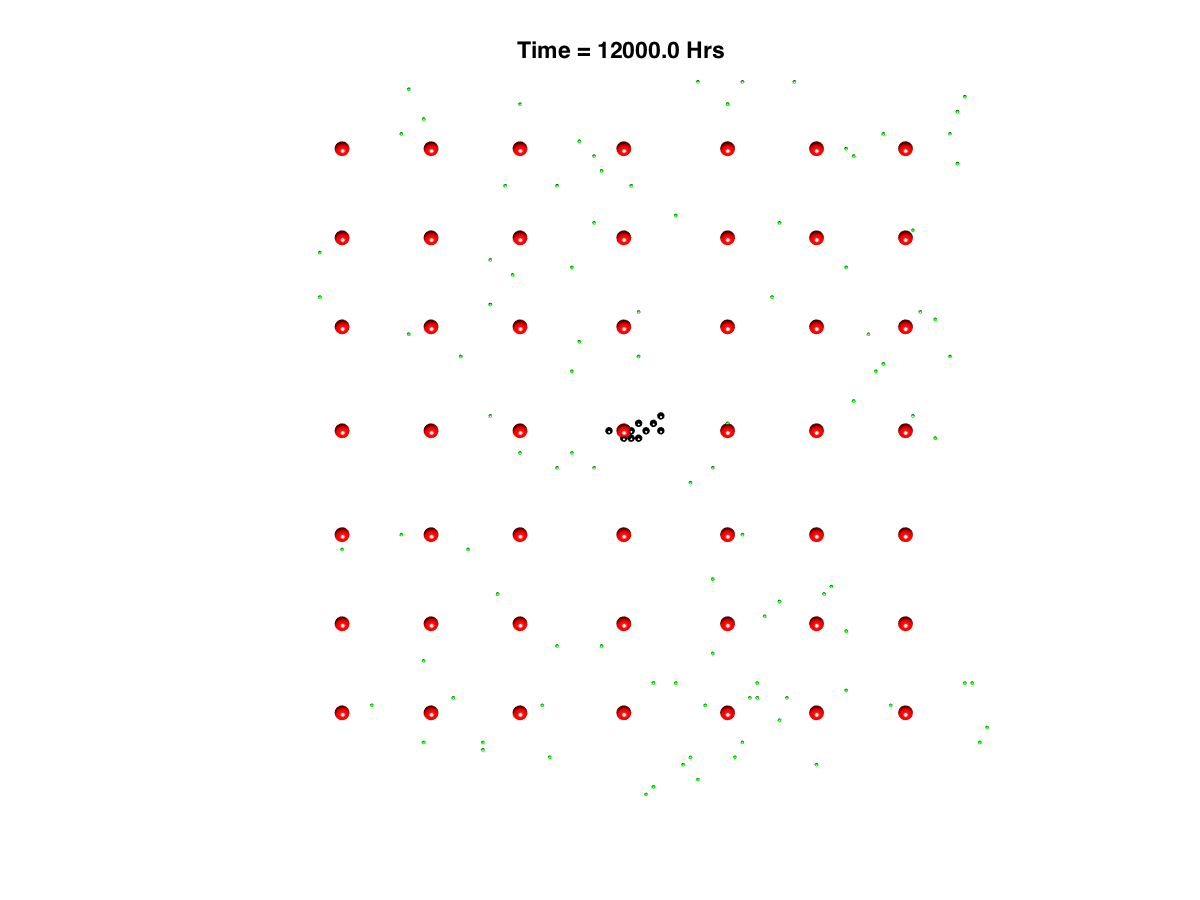

Supplement: Supplementary Data S1 — Supplementary Raw Research Data. This is open data under the CC BY license http://creativecommons.org/licenses/by/4.0/ [file mmc1.zip › Supplementary material/Figures 7-10/Figure7bi.png]

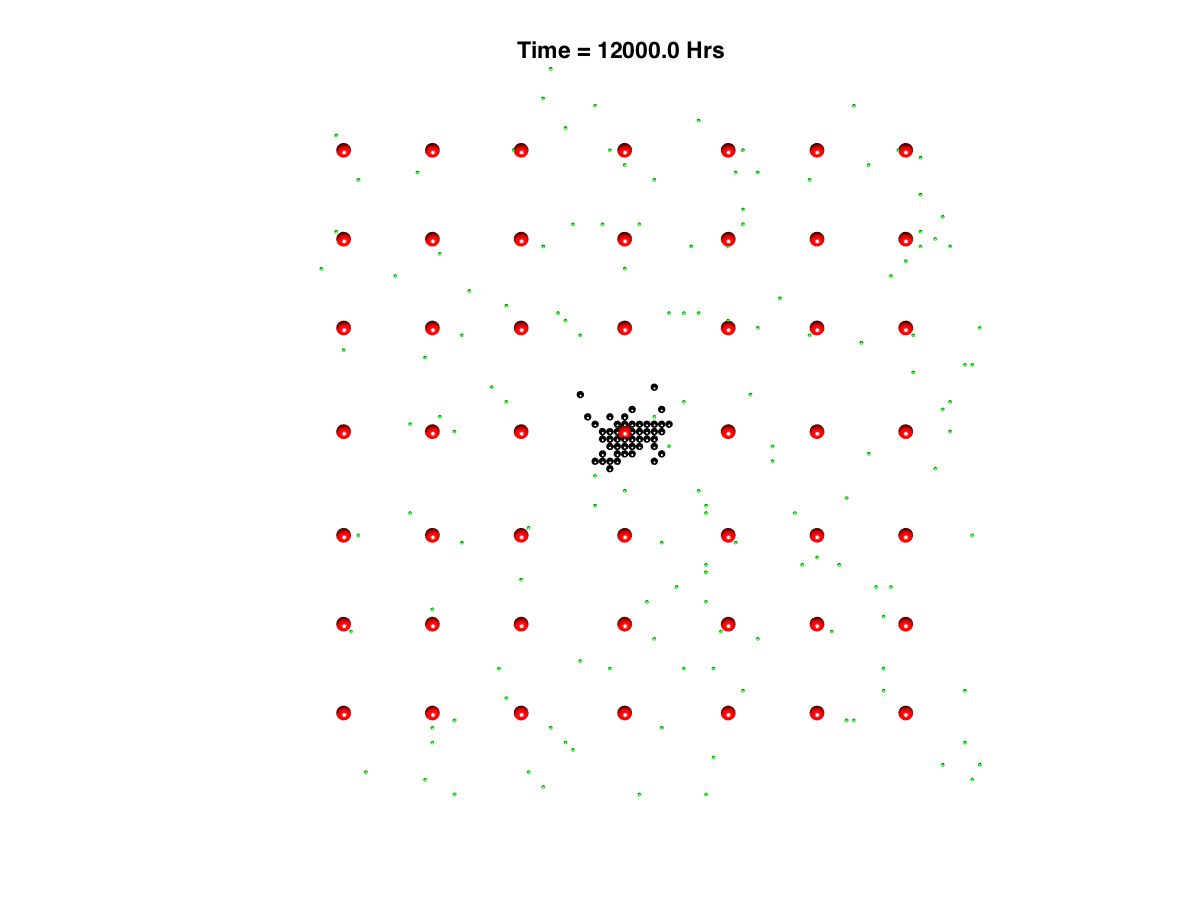

Supplement: Supplementary Data S1 — Supplementary Raw Research Data. This is open data under the CC BY license http://creativecommons.org/licenses/by/4.0/ [file mmc1.zip › Supplementary material/Figures 7-10/Figure7bii.png]

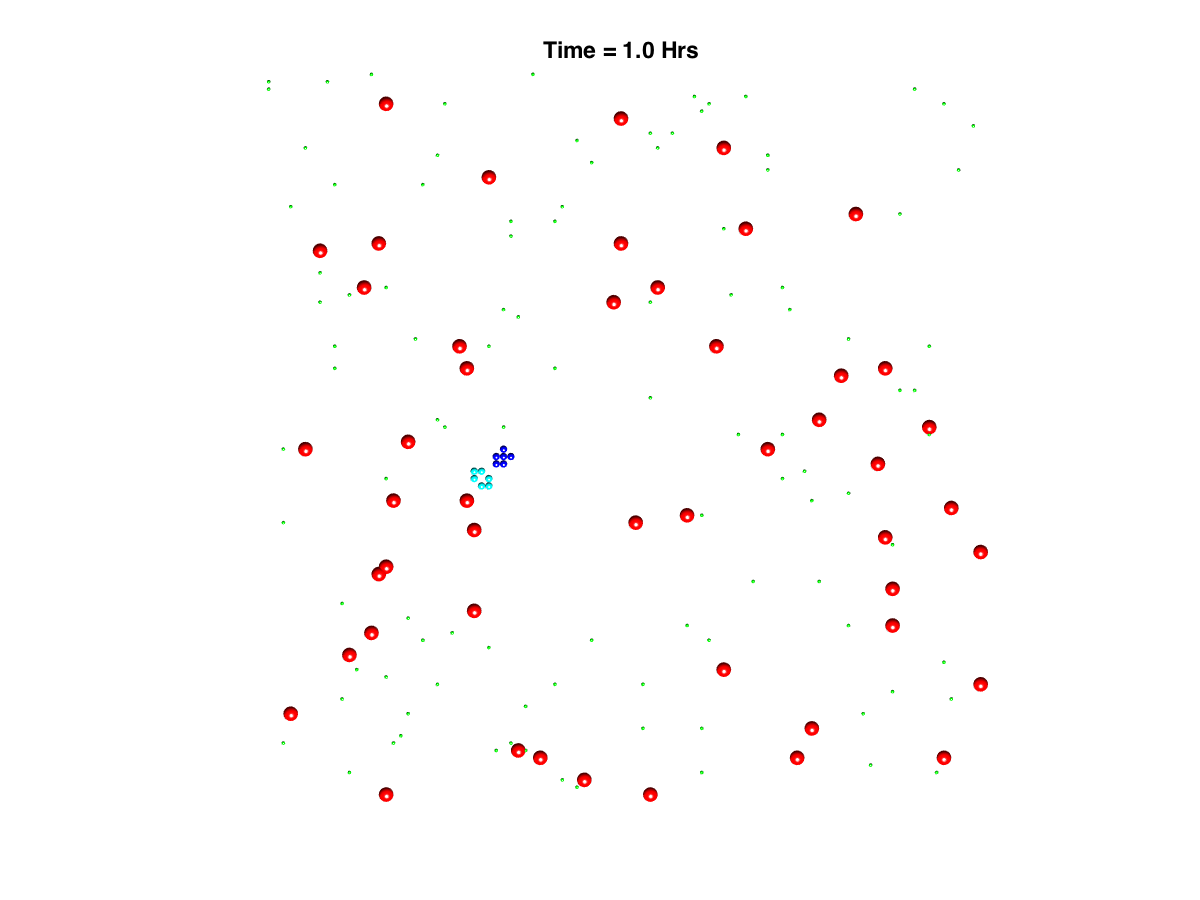

Supplement: Supplementary Data S1 — Supplementary Raw Research Data. This is open data under the CC BY license http://creativecommons.org/licenses/by/4.0/ [file mmc1.zip › Supplementary material/Figures 7-10/Figure8ai.png]

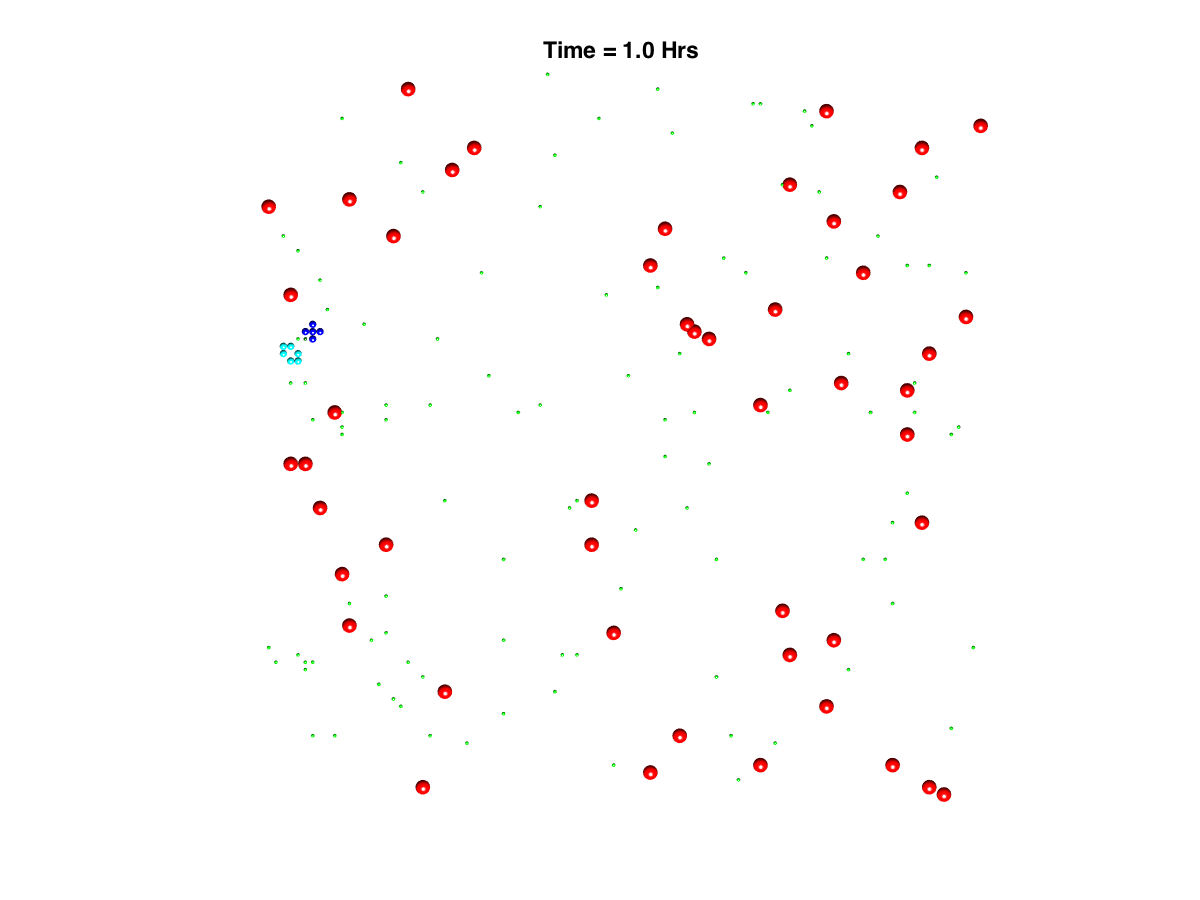

Supplement: Supplementary Data S1 — Supplementary Raw Research Data. This is open data under the CC BY license http://creativecommons.org/licenses/by/4.0/ [file mmc1.zip › Supplementary material/Figures 7-10/Figure8aii.png]

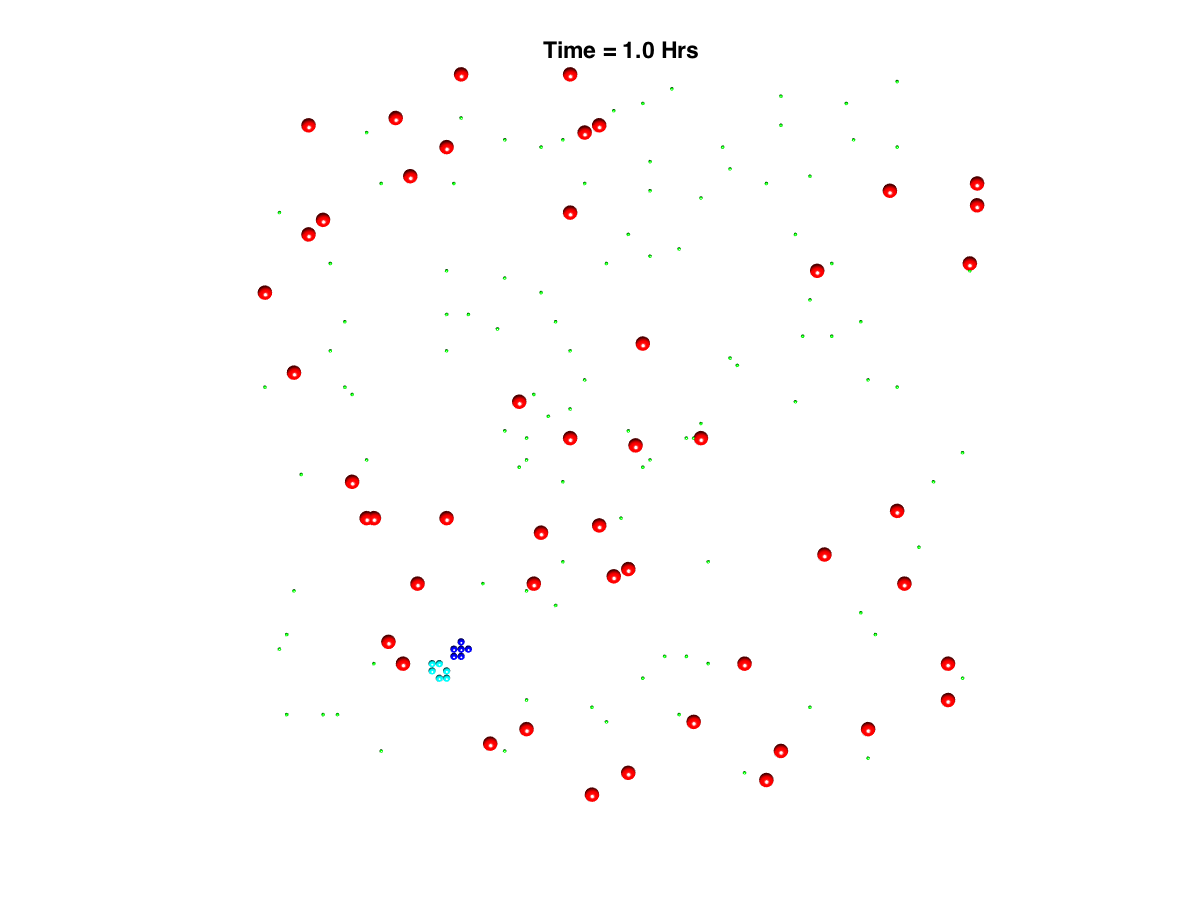

Supplement: Supplementary Data S1 — Supplementary Raw Research Data. This is open data under the CC BY license http://creativecommons.org/licenses/by/4.0/ [file mmc1.zip › Supplementary material/Figures 7-10/Figure8aiii.png]

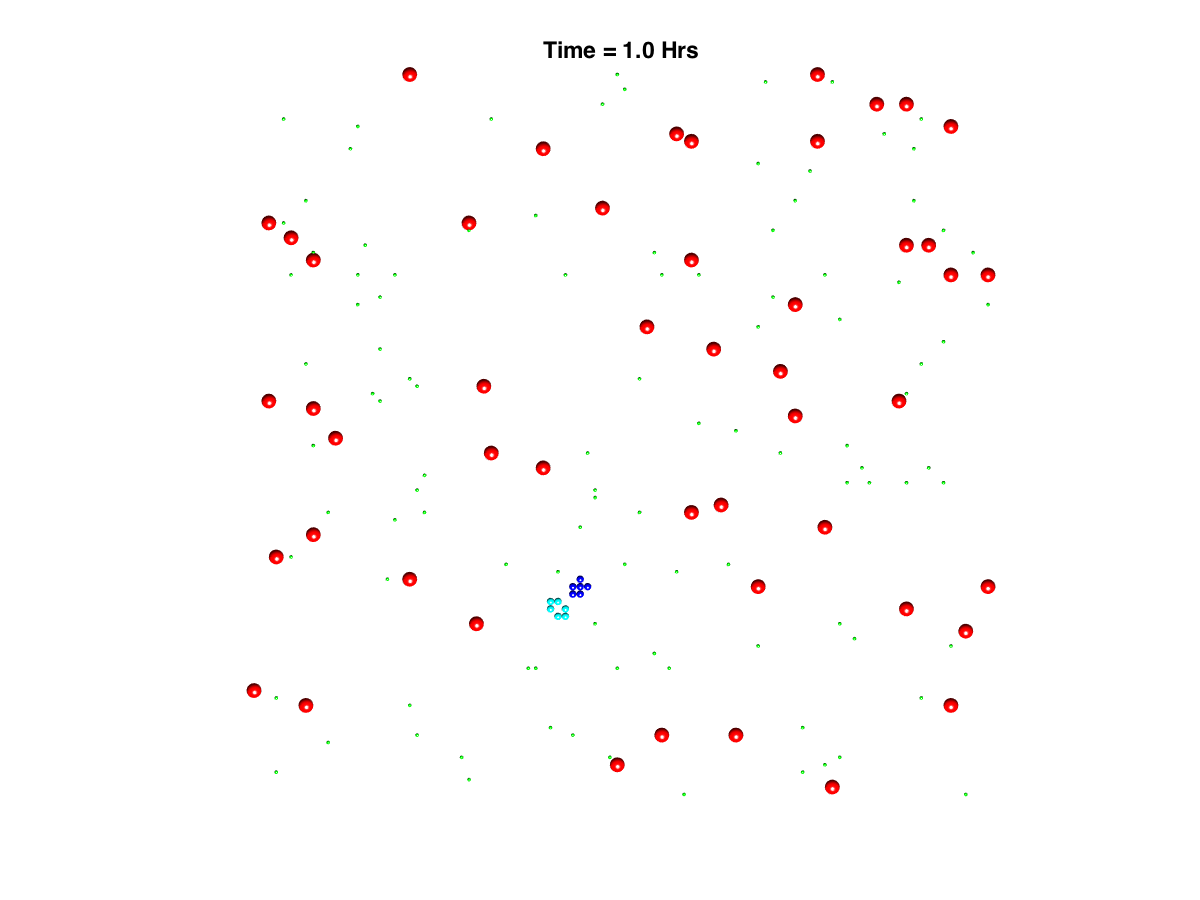

Supplement: Supplementary Data S1 — Supplementary Raw Research Data. This is open data under the CC BY license http://creativecommons.org/licenses/by/4.0/ [file mmc1.zip › Supplementary material/Figures 7-10/Figure8aiv.png]

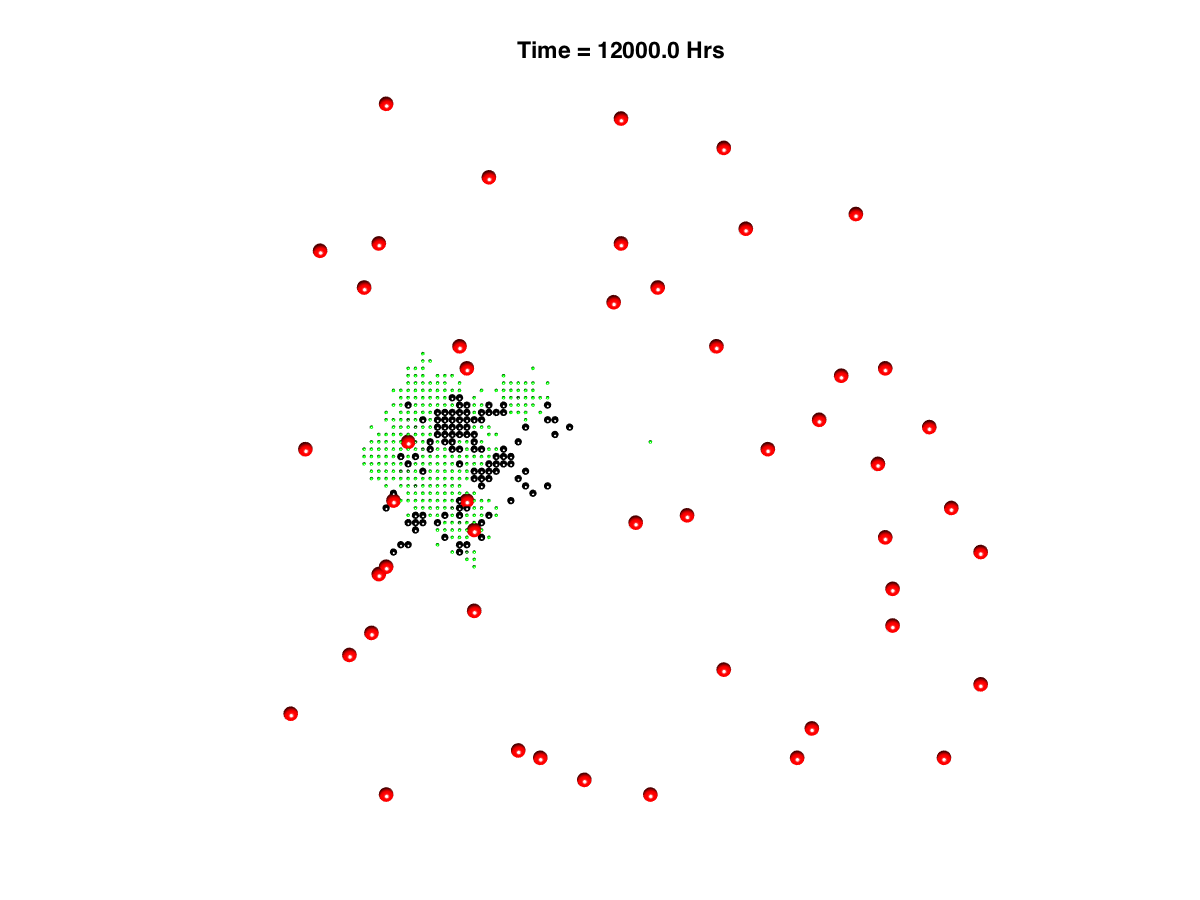

Supplement: Supplementary Data S1 — Supplementary Raw Research Data. This is open data under the CC BY license http://creativecommons.org/licenses/by/4.0/ [file mmc1.zip › Supplementary material/Figures 7-10/Figure8bi.png]

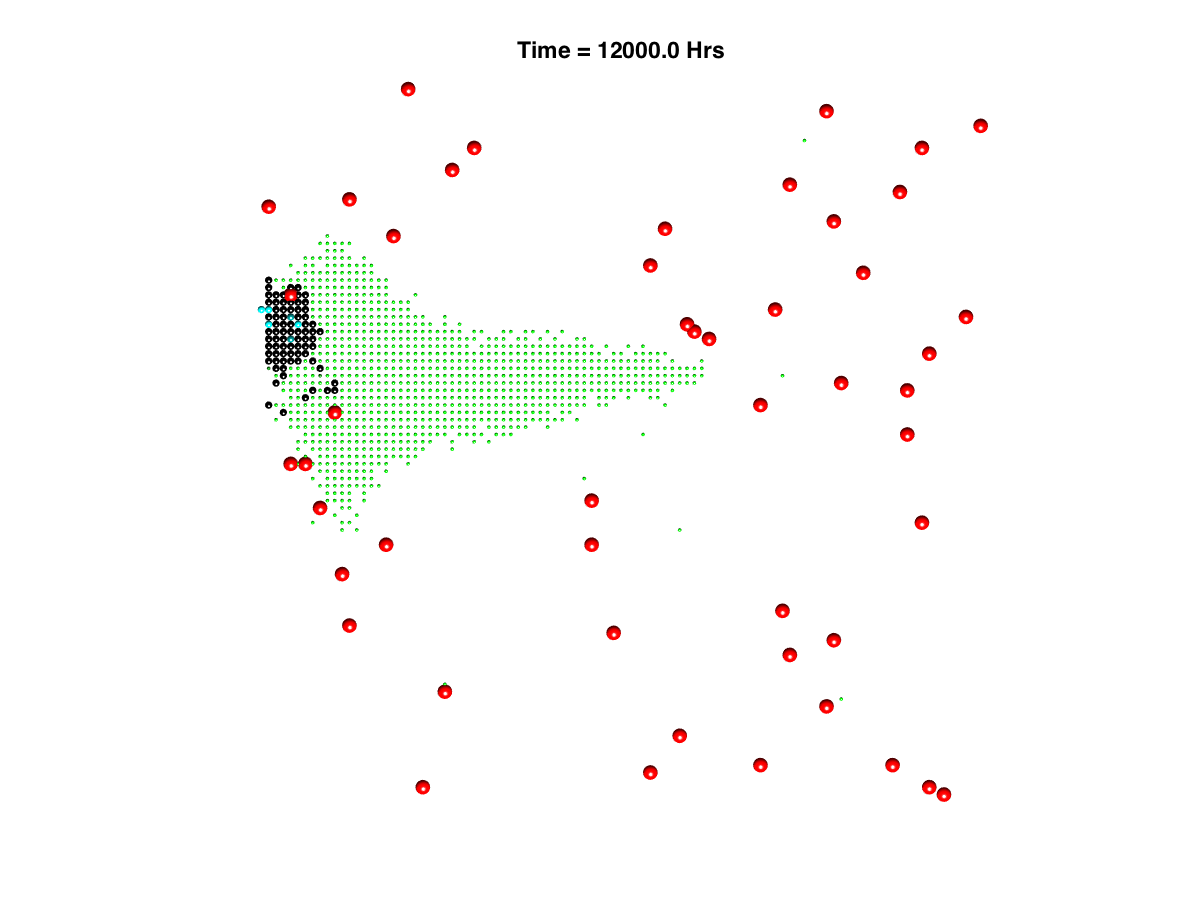

Supplement: Supplementary Data S1 — Supplementary Raw Research Data. This is open data under the CC BY license http://creativecommons.org/licenses/by/4.0/ [file mmc1.zip › Supplementary material/Figures 7-10/Figure8bii.png]

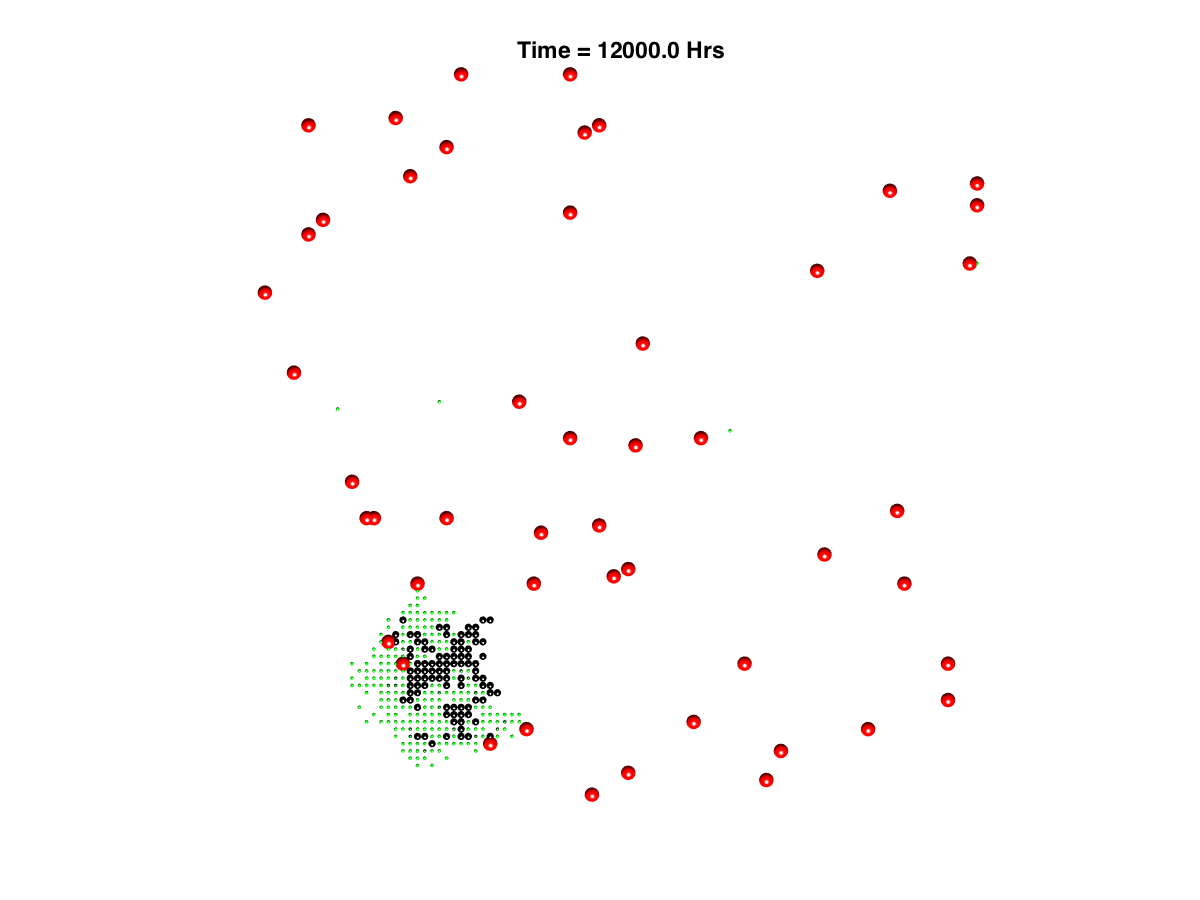

Supplement: Supplementary Data S1 — Supplementary Raw Research Data. This is open data under the CC BY license http://creativecommons.org/licenses/by/4.0/ [file mmc1.zip › Supplementary material/Figures 7-10/Figure8biii.png]

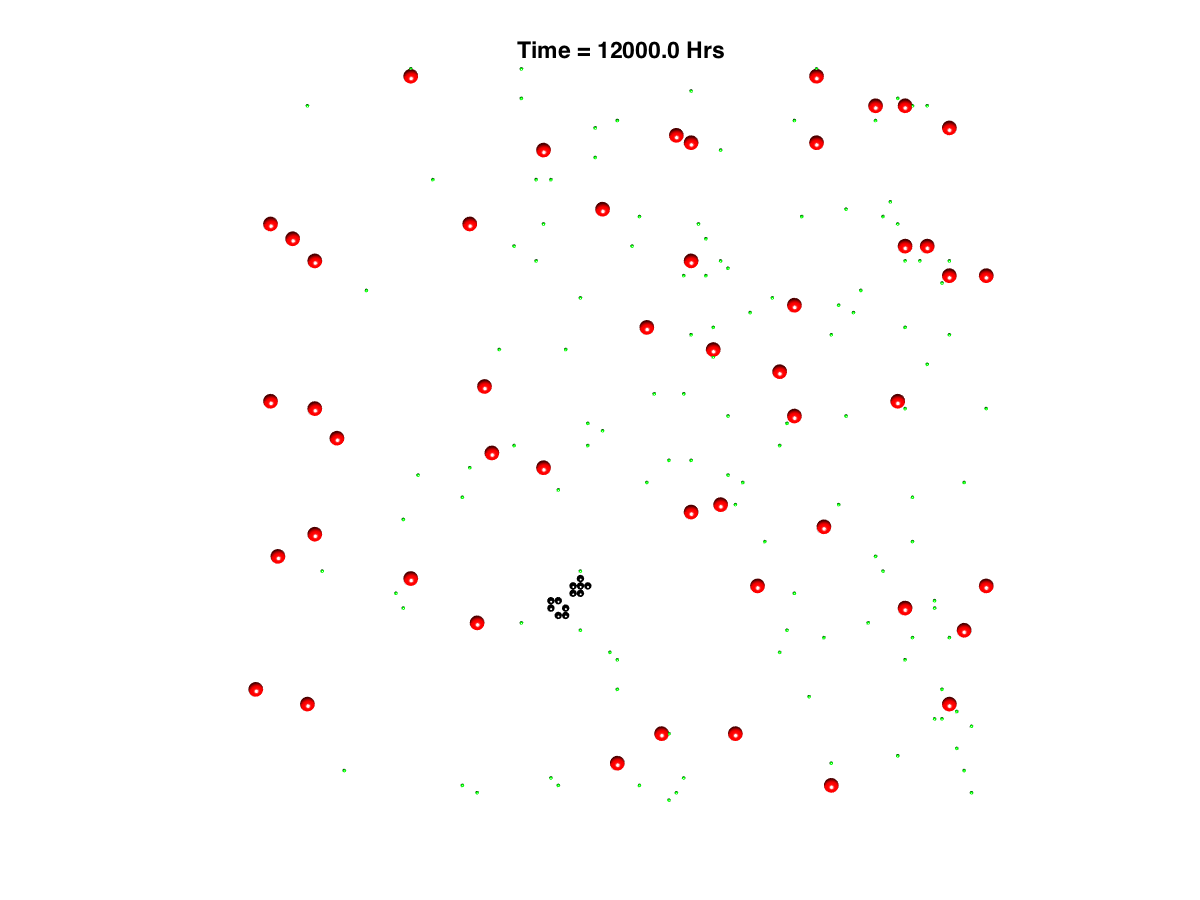

Supplement: Supplementary Data S1 — Supplementary Raw Research Data. This is open data under the CC BY license http://creativecommons.org/licenses/by/4.0/ [file mmc1.zip › Supplementary material/Figures 7-10/Figure8biv.png]

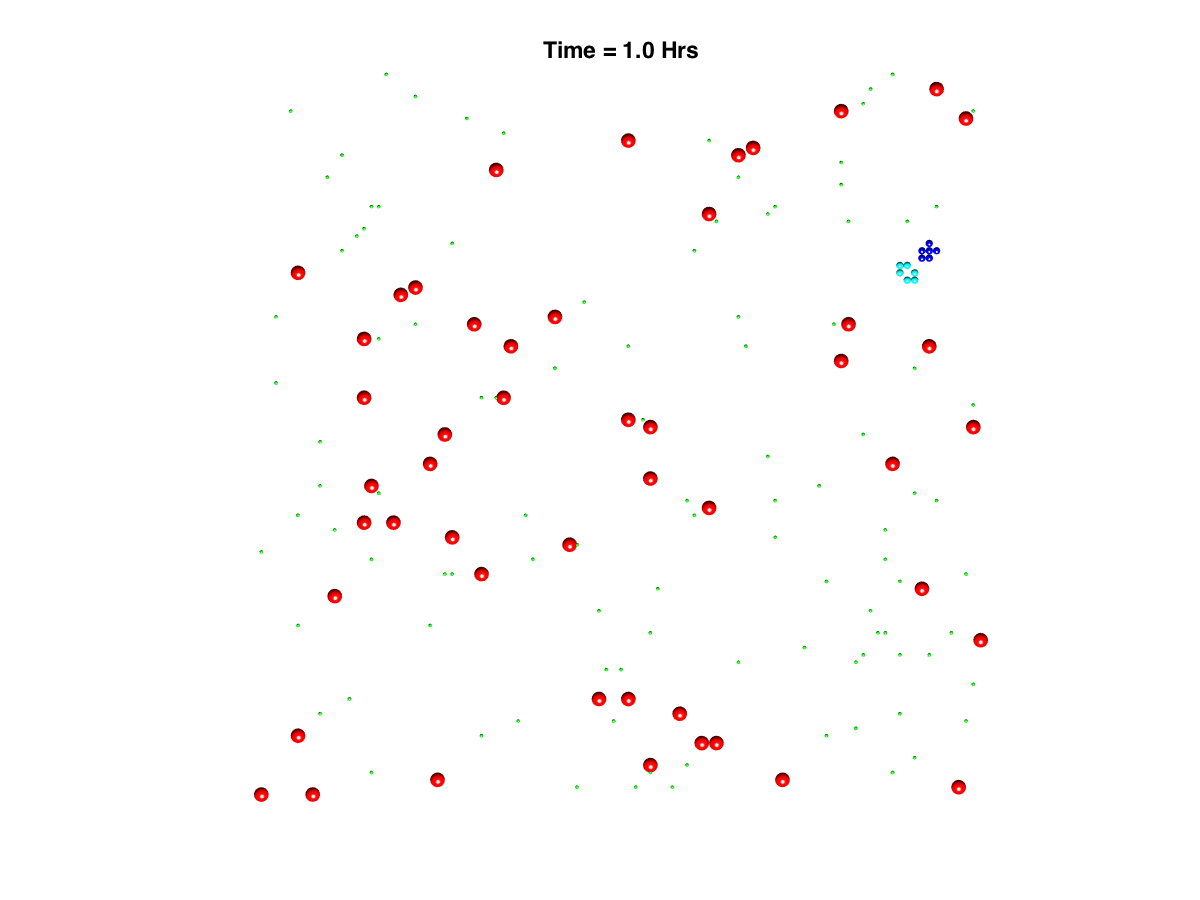

Supplement: Supplementary Data S1 — Supplementary Raw Research Data. This is open data under the CC BY license http://creativecommons.org/licenses/by/4.0/ [file mmc1.zip › Supplementary material/Figures 7-10/Figure9ai.png]

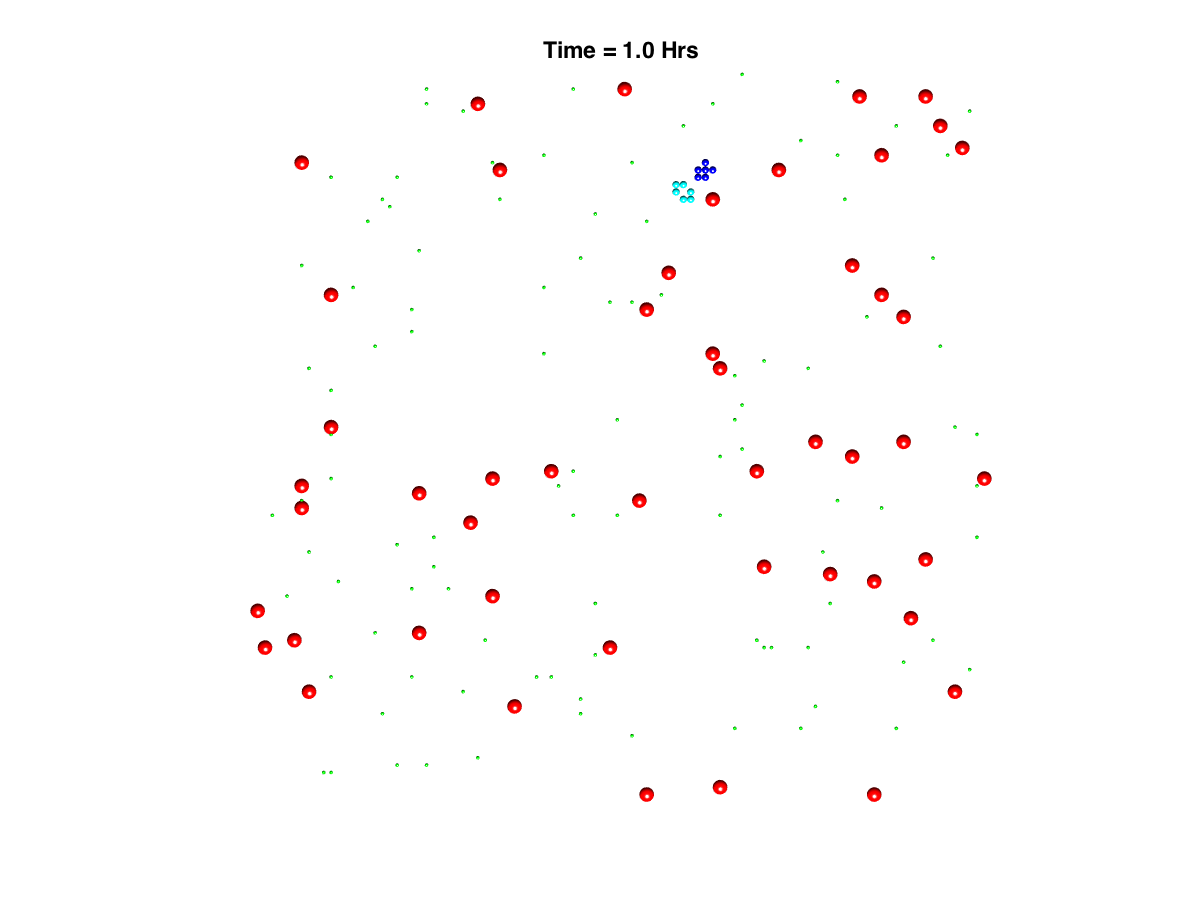

Supplement: Supplementary Data S1 — Supplementary Raw Research Data. This is open data under the CC BY license http://creativecommons.org/licenses/by/4.0/ [file mmc1.zip › Supplementary material/Figures 7-10/Figure9aii.png]

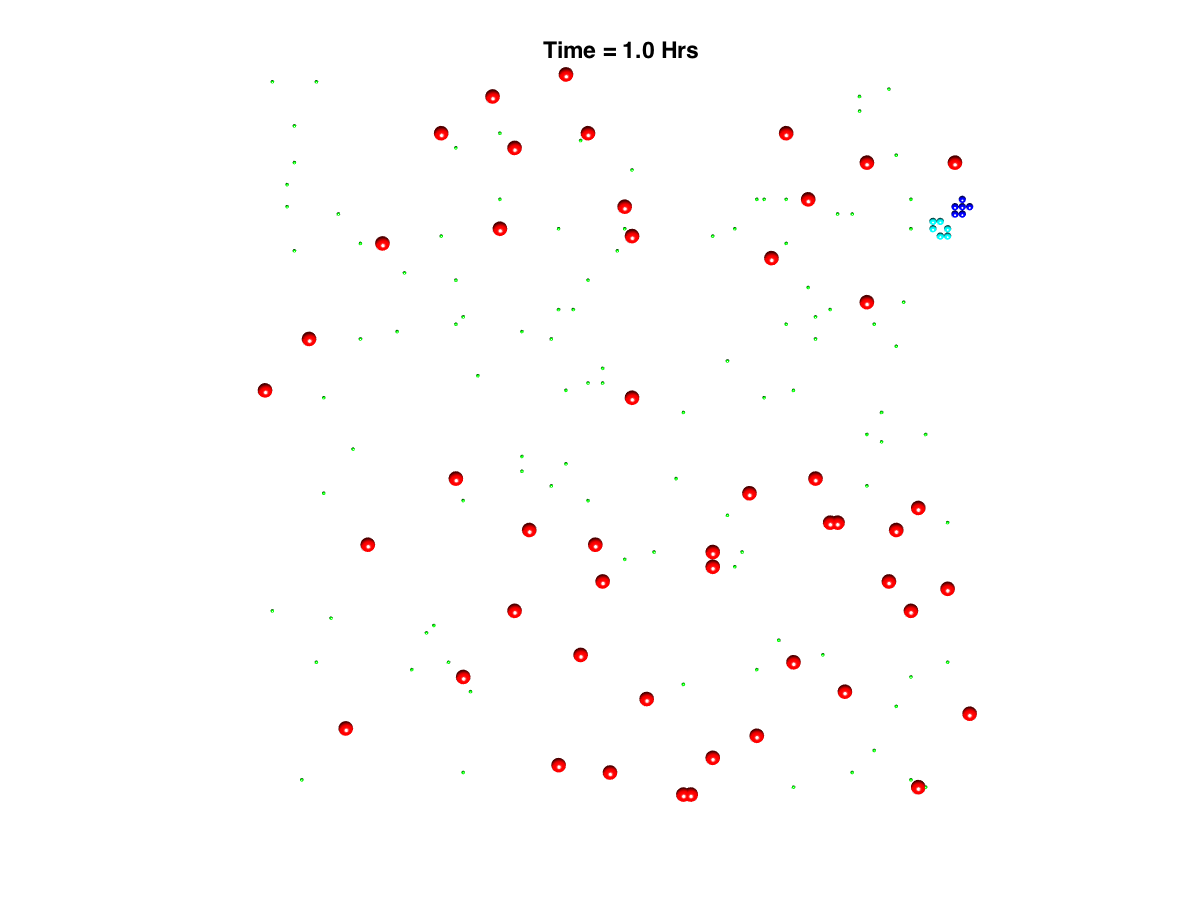

Supplement: Supplementary Data S1 — Supplementary Raw Research Data. This is open data under the CC BY license http://creativecommons.org/licenses/by/4.0/ [file mmc1.zip › Supplementary material/Figures 7-10/Figure9aiii.png]

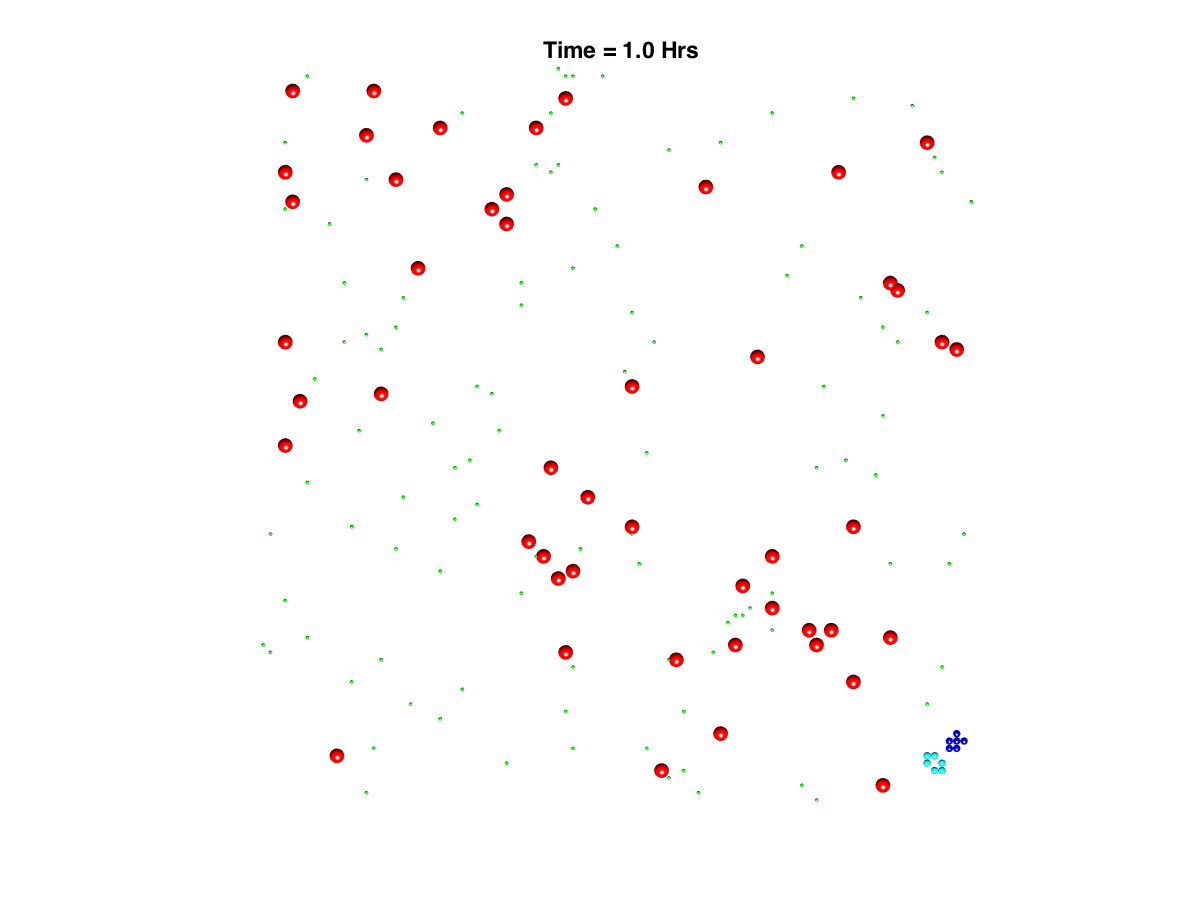

Supplement: Supplementary Data S1 — Supplementary Raw Research Data. This is open data under the CC BY license http://creativecommons.org/licenses/by/4.0/ [file mmc1.zip › Supplementary material/Figures 7-10/Figure9aiv.png]

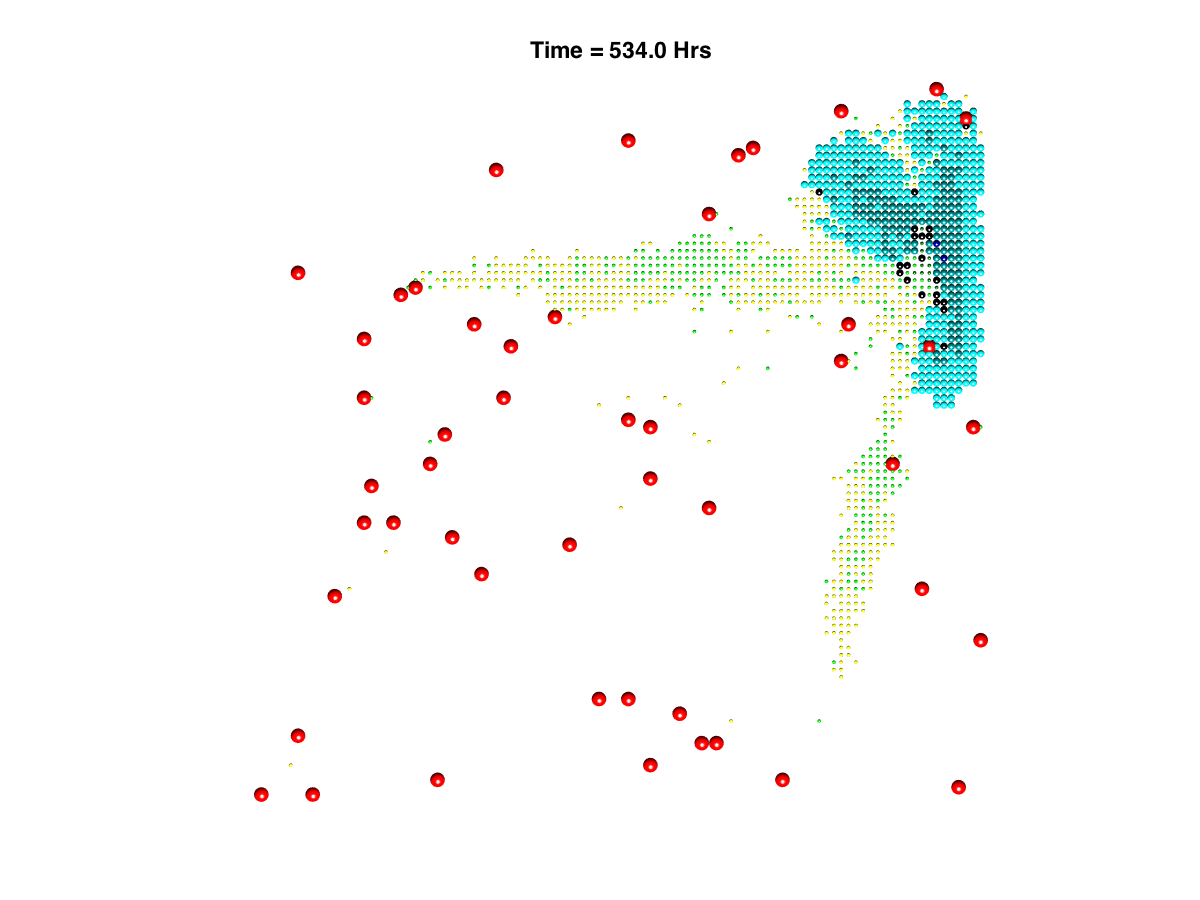

Supplement: Supplementary Data S1 — Supplementary Raw Research Data. This is open data under the CC BY license http://creativecommons.org/licenses/by/4.0/ [file mmc1.zip › Supplementary material/Figures 7-10/Figure9bi.png]

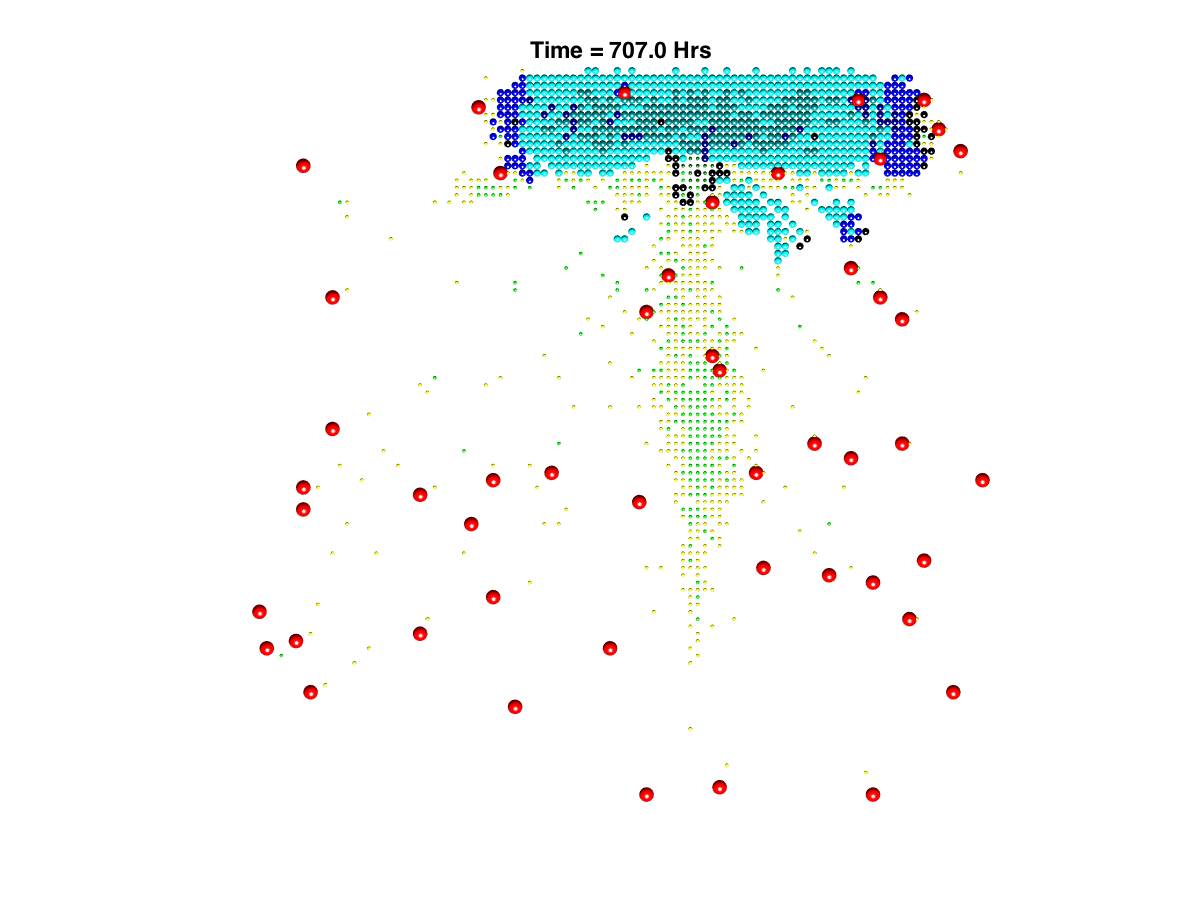

Supplement: Supplementary Data S1 — Supplementary Raw Research Data. This is open data under the CC BY license http://creativecommons.org/licenses/by/4.0/ [file mmc1.zip › Supplementary material/Figures 7-10/Figure9bii.png]

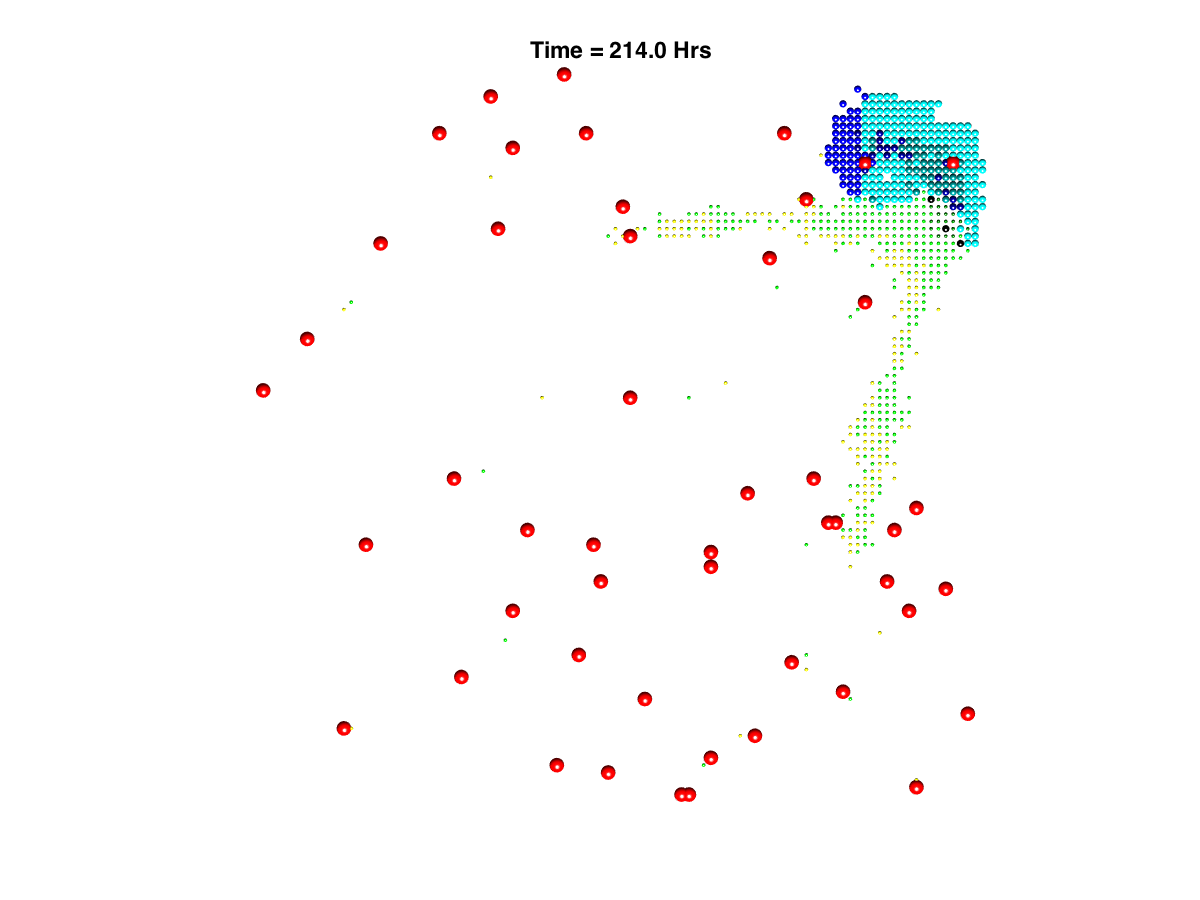

Supplement: Supplementary Data S1 — Supplementary Raw Research Data. This is open data under the CC BY license http://creativecommons.org/licenses/by/4.0/ [file mmc1.zip › Supplementary material/Figures 7-10/Figure9biii.png]

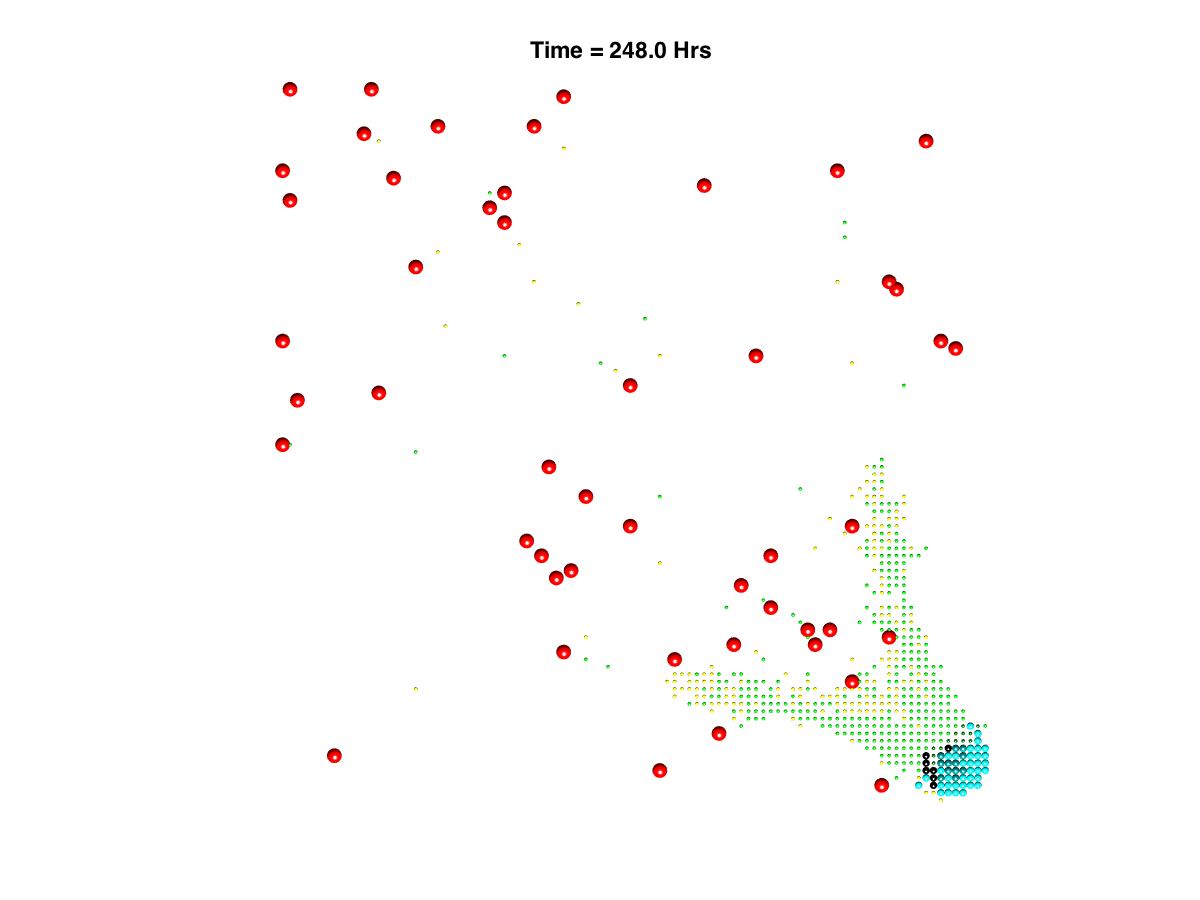

Supplement: Supplementary Data S1 — Supplementary Raw Research Data. This is open data under the CC BY license http://creativecommons.org/licenses/by/4.0/ [file mmc1.zip › Supplementary material/Figures 7-10/Figure9biv.png]

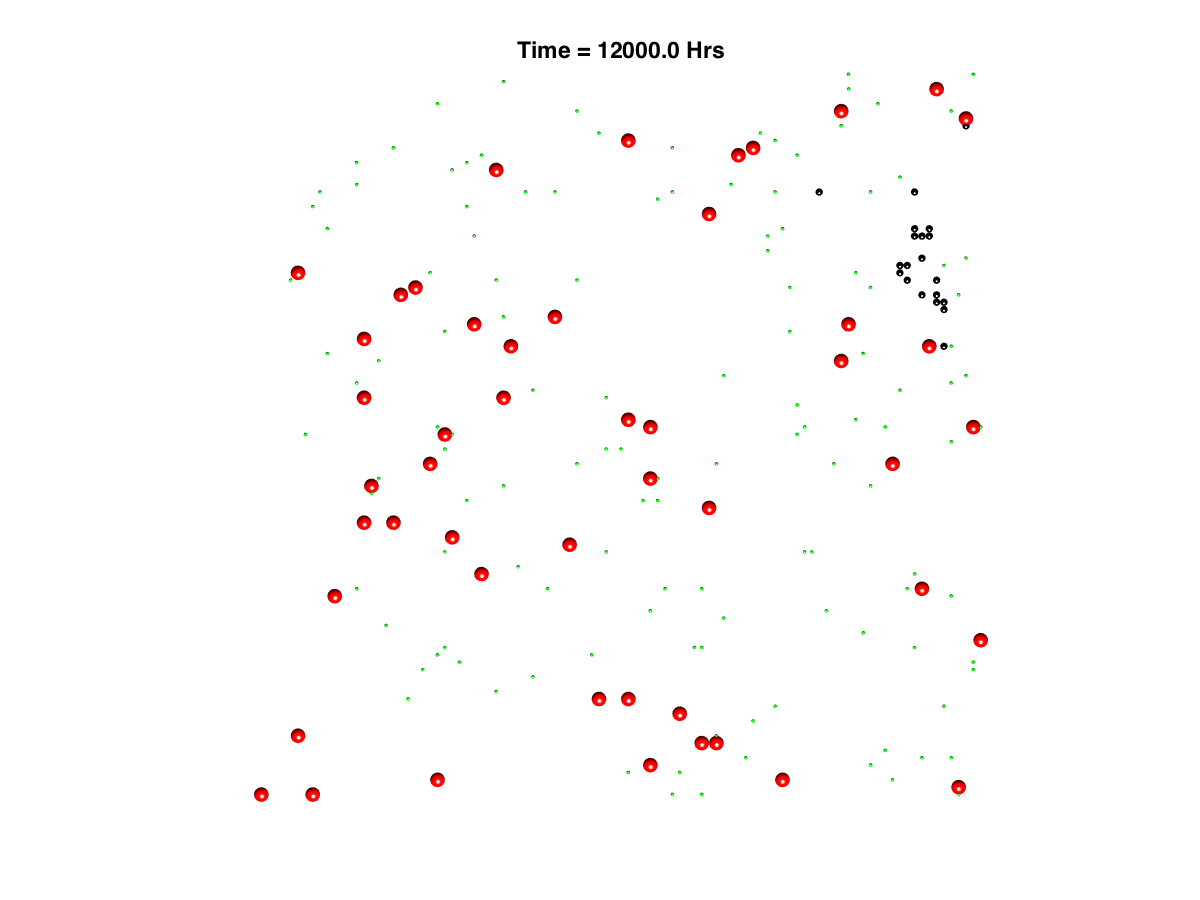

Supplement: Supplementary Data S1 — Supplementary Raw Research Data. This is open data under the CC BY license http://creativecommons.org/licenses/by/4.0/ [file mmc1.zip › Supplementary material/Figures 7-10/Figure9ci.png]

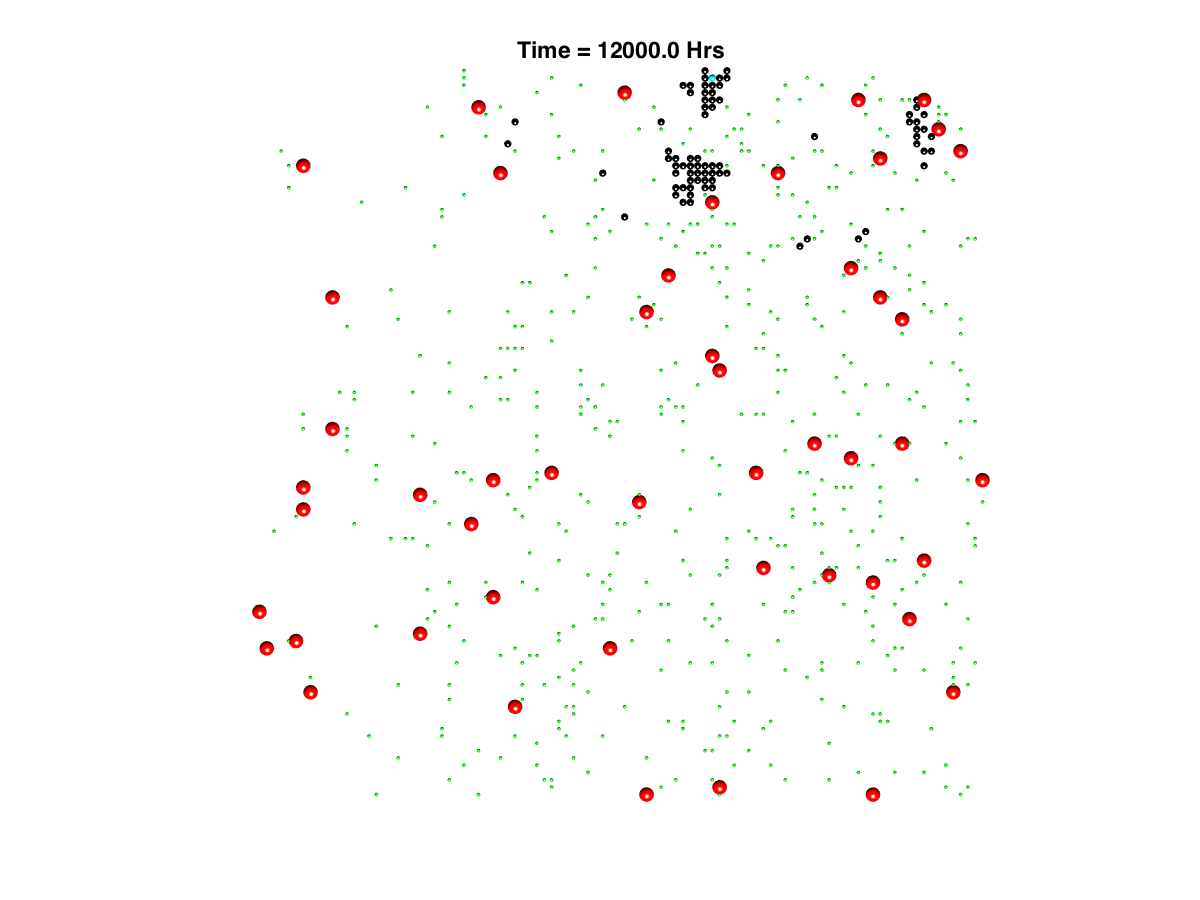

Supplement: Supplementary Data S1 — Supplementary Raw Research Data. This is open data under the CC BY license http://creativecommons.org/licenses/by/4.0/ [file mmc1.zip › Supplementary material/Figures 7-10/Figure9cii.png]

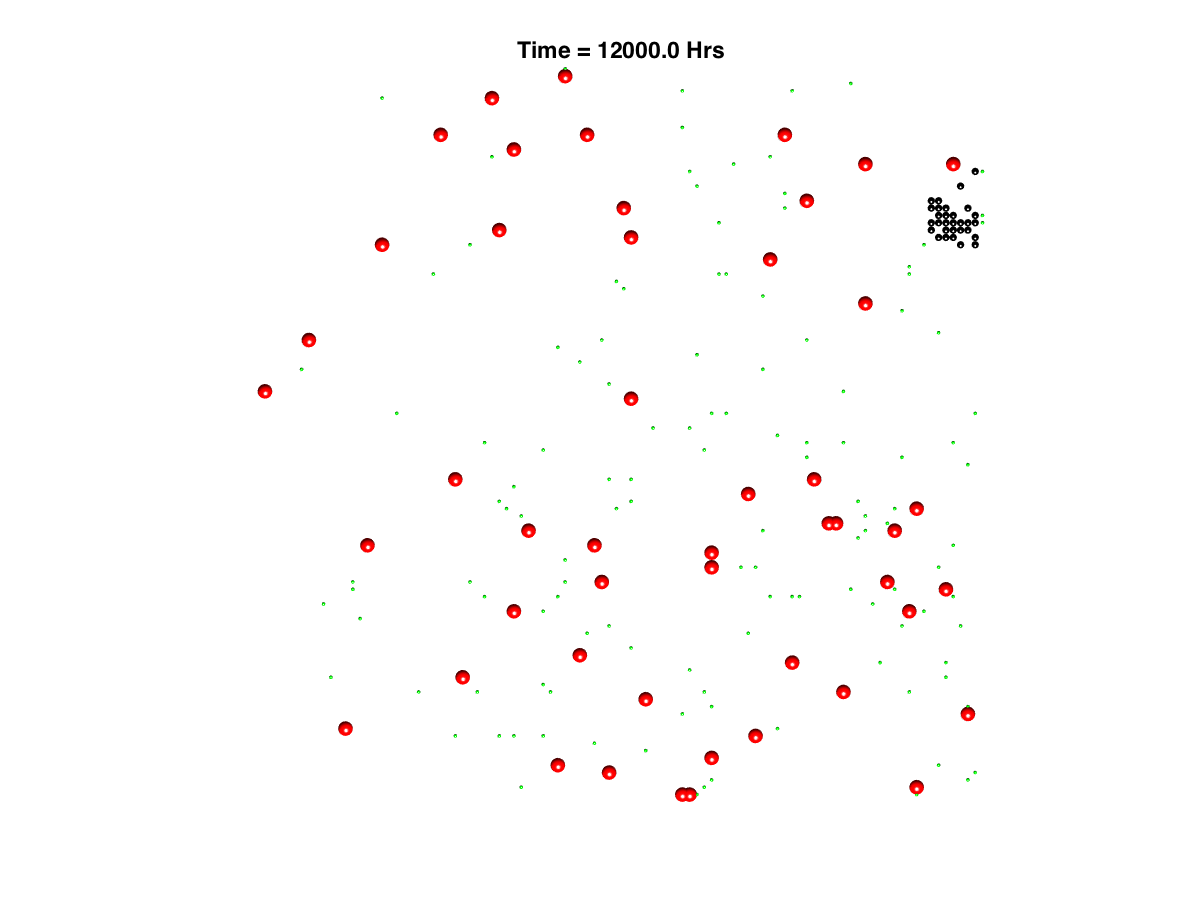

Supplement: Supplementary Data S1 — Supplementary Raw Research Data. This is open data under the CC BY license http://creativecommons.org/licenses/by/4.0/ [file mmc1.zip › Supplementary material/Figures 7-10/Figure9ciii.png]

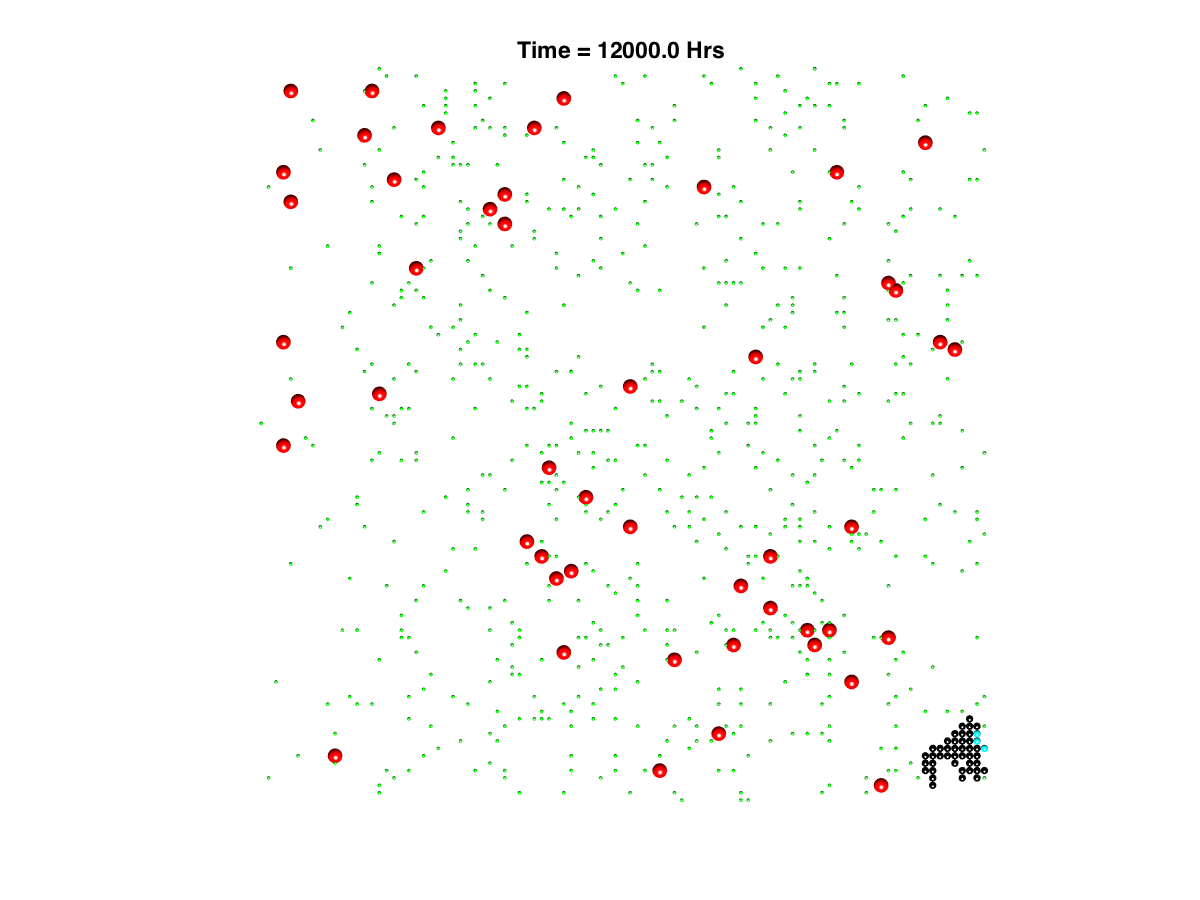

Supplement: Supplementary Data S1 — Supplementary Raw Research Data. This is open data under the CC BY license http://creativecommons.org/licenses/by/4.0/ [file mmc1.zip › Supplementary material/Figures 7-10/Figure9civ.png]
